# Deciphering single-cell landscape unravels cell-type-specific functional roles of RNA m^6^A modification in atherosclerosis

**DOI:** 10.7150/thno.104179

**Published:** 2025-03-29

**Authors:** Xiaorui Ping, Xiaoyun Liang, Wenlu Xing, Saiqi Wang, Fengcongzhe Gong, Yaqi Cheng, Songqi Duan, Xueqi Lv, Xueying Li, Tianli Zhang, Chunxiao Chen, Yuxin Zhang, Chengzhu Yuan, Shangyu Liu, Gang Liu, Baofa Sun

**Affiliations:** 1 State Key Laboratory of Medicinal Chemical Biology, Frontiers Science Center for Cell Responses, College of Life Sciences, Nankai University, Tianjin 300071, China.; 2 Department of Cardiology, The First Hospital of Hebei Medical University, Shijiazhuang, Hebei 050031, China.; 3 Hebei Key Laboratory of Cardiac Injury Repair Mechanism Study, Shijiazhuang, Hebei 050031, China.; 4 Hebei Engineering Research Center of Intelligent Medical Clinical Application, Shijiazhuang, Hebei 050031, China.; 5 Hebei International Joint Research Center for Structural Heart Disease, Shijiazhuang, Hebei 050031, China.; 6 College of Food Science, Sichuan Agricultural University, Sichuan University, Chengdu 610000, China.

**Keywords:** Single-cell, m^6^A, Atherosclerosis, Smooth muscle cell, Endothelial cell, Macrophage

## Abstract

**Background**: Atherosclerosis is a chronic inflammatory disease that is the major cause of mortality worldwide. Although several studies have assessed the function of m^6^A (N^6^-methyladenosine) modification in atherosclerosis, its regulatory mechanism at the single-cell level remains unclear. This study provides a comprehensive single-cell atlas of m^6^A modification regulating cell-type-specific functions in atherosclerosis.

**Methods**: We analyzed single-cell sequencing data derived from atherosclerosis patients to elucidate the influence of m^6^A modification on diverse cell types. We demonstrated the potential regulatory functions of m^6^A regulators across various cell types and key transcription factors involved. Furthermore, we discovered m^6^A regulators mediated intercellular communication in important biological processes. *In vitro* experiments were conducted to further investigate the effects of ALKBH5, WTAP and METTL3 on atherosclerosis.

**Results**: *ALKBH5* upregulated in endothelial cells induced cell proliferation and migration involved in sprouting angiogenesis. In smooth muscle cells, upregulation of *WTAP* enhanced proliferation, migration and phenotypic transformation. Upregulation of *METTL3* and *YTHDF2* promoted macrophage activation and differentiation. Furthermore, we identified abnormally activated transcription factors could regulate m^6^A regulators in a cell-type-specific manner. Moreover, we revealed that m^6^A regulators were implicated in dysregulated intercellular communication in atherosclerosis. And a series of experimental validations supported the conclusion that m^6^A regulators exert cell-type-specific regulatory functions.

**Conclusion**: Our study provided evidence for the roles of ALKBH5, WTAP and METTL3 in orchestrating atherosclerotic cell-type-specific functions, representing promising targets for precision medicine.

## Introduction

Cardiovascular diseases (CVDs) are responsible for the increased morbidity and mortality throughout the world. The development and rupture of plaques is a major cause of CVDs, resulting in complications such as myocardial infarction and stroke 1. The progression of atherosclerotic lesions is highly intricate, encompassing multiple cell types and molecular mechanisms 2-4. The alteration of endothelial cell (EC) phenotype is a critical component in the initiation of atherosclerosis. Concurrently, hemodynamic factors within the vascular system drive the activation of ECs 5. Abnormal vascular regions increase the expression of EC-related inflammatory genes, intensifying leukocytes recruitment and fueling the inflammatory cascade spread 4. In addition, smooth muscle cells (SMCs) within the plaque are capable of enhancing plaque stability by forming and thickening the fibrous cap. However, SMCs can also exist in inflammatory and calcified states that contribute to plaque instability 6. Several anti-atherosclerotic therapeutic drugs have been widely used in clinical practice, and the most representative of which are statins 7. Although these drugs are effective in controlling patients' lipid levels, some patients still have a high residual cardiovascular risk, which may result in recurrent clinical events 8. To solve clinical issues and develop effective treatments for atherosclerosis, we must understand the mechanisms behind its development.

Single-cell RNA sequencing (scRNA-seq) reveals detailed transcriptional changes, helping to investigate cellular makeup and mechanisms in atherosclerosis 9. A review has been conducted on the advancements in single-cell analysis of atherosclerosis, elucidating the heterogeneity of cell types within the disease 10. Atherosclerosis progression involves abnormal processes like monocyte and lymphocyte recruitment, SMC migration and proliferation, and EC dysfunction 2. Through single-cell technology, we can gain a profound understanding of the diversified situations of these key cell types 11-17. Lin *et al.* found a population of proliferating CX3CR1^+^ monocytes with stem cell-like characteristics in both progression and regression plaques 15. Pan *et al.* showed that in atherosclerosis, SMCs transition to an intermediate cell state known as “SEM” cells, which have multi-directional differentiation potential and can differentiate into macrophage-like and fibrochondrocyte-like cells 18. Zhao *et al.* identified three EC clusters expressing mesenchymal markers by scRNA-seq, indicating the occurrence of endothelial-to-mesenchymal transition in mouse hearts 19. Overall, scRNA-seq plays an important role in the elucidation of the underlying pathological mechanisms of atherosclerosis in different cell types.

In recent years, studies on atherosclerosis have advanced to explore the genetic and epigenetic levels 20. A form of epigenetic modification, m^6^A (N^6^-methyladenosine) modification, plays an important role in the development of atherosclerosis 21-25. For example, Li *et al.* discovered that METTL3 interacts with epidermal growth factor receptor (EGFR), which has the potential to impact atherosclerosis 26,27. The m^6^A modification of EGFR 3'UTR accelerated its mRNA degradation, while double mutation in the EGFR 3'UTR eliminated METTL3-induced luciferase activity. The mechanism by which METTL3 attenuated the progression of ECs via the m^6^A-dependent stability of EGFR mRNA was elucidated 27. Jian *et al.* found that the downregulation of *METTL14* expression inhibited EC inflammation and the progression of atherosclerosis 28. Mo *et al.* demonstrated that FTO inhibited foam cells formation by controlling cholesterol efflux transporters and scavenger receptors, while inhibiting *FTO* expression could promote the formation of atherosclerotic plaques 29. However, further studies are required to understand the regulatory mechanisms of m^6^A regulators in atherosclerosis at the single-cell level.

In this study, we elucidated the potential regulatory mechanism of m^6^A modification in key atherosclerotic cell types. We found cell-type-specific expression patterns of m^6^A regulators in atherosclerosis. Upregulated *WTAP* expression promoted SMC proliferation and migration, while *ALKBH5* upregulation in ECs induced cell proliferation and migration involved in sprouting angiogenesis. *METTL3* and *YTHDF2* enhanced macrophage activation and differentiation. We also identified a potential regulatory role of WTAP in SMC phenotypic transformation. Additionally, we uncovered cell-type-specific transcription factors (TFs) that regulate the expression of m^6^A regulators. Moreover, we revealed the regulatory roles of m^6^A regulators in cell-cell communication and several significant ligand-receptor pairs in atherosclerosis. The experimental results confirmed the potential regulatory roles of ALKBH5, WTAP and METTL3 in angiogenesis, cell proliferation, migration and inflammatory process. Altogether, our findings offer a detailed single-cell perspective on the regulatory role of m^6^A regulators in atherosclerosis, providing new pathophysiological insights and aiding therapeutic strategy development.

## Results

### Cell-type-specific expression patterns of m^6^A regulators and their potential roles in biological processes of atherosclerosis

**Figure 1:**
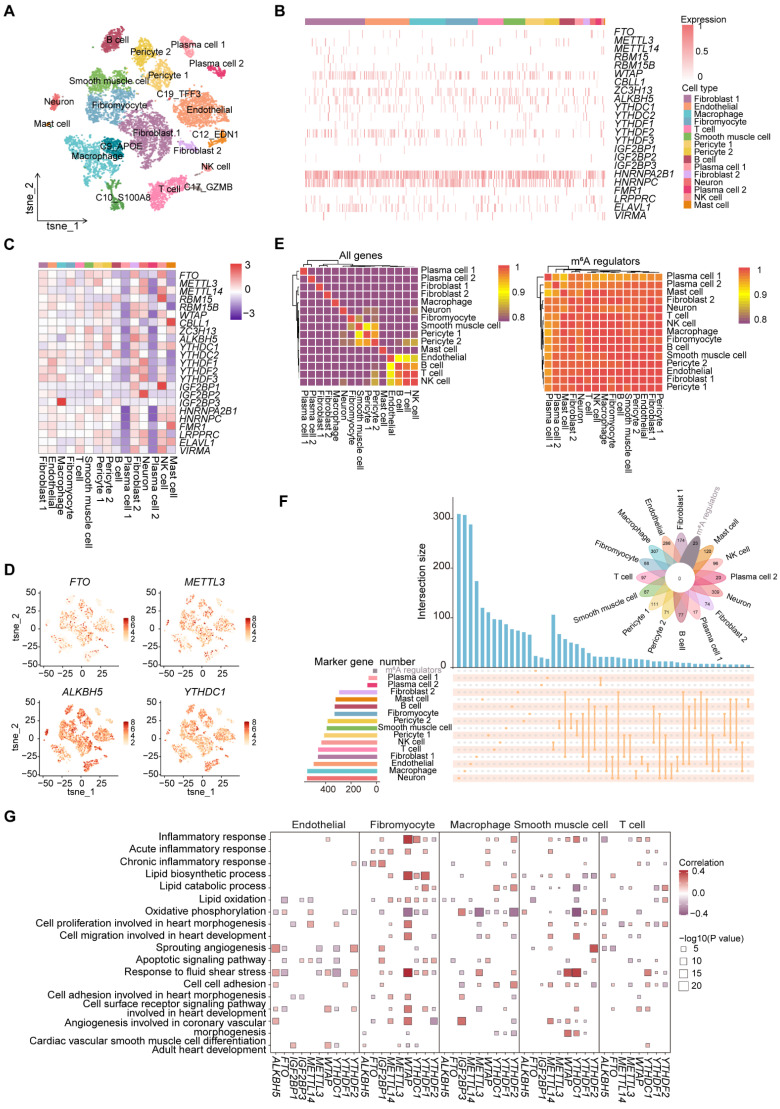
** Expression profile of m^6^A regulators in atherosclerotic cell types.** (A) The t-SNE projection of 11,722 single cells from atherosclerotic patients, with 20 main cell clusters shown in different colors. Each dot corresponds to one single cell, colored according to cell type. (B) Distribution of m^6^A regulators across different identified cell types. (C) Heatmap showing the average expression of m^6^A regulators in major cell types. (D) T-SNE projections of 20 clusters, with each cell colored by the relative normalized expression of m^6^A regulators. (E) Heatmap showing the expression patterns of different cell types based on all genes (left) and m^6^A regulators (right). (F) Upset and venn plots showing the intersection between marker genes of different cell types and m^6^A regulators. (G) Correlation analysis between signatures of disease-related pathways and the expression levels of m^6^A regulators in key cell types. *P* value was calculated by Pearson correlation.

To delve deeper into the potential regulatory roles of m^6^A regulators in atherosclerosis, we obtained the scRNA-seq expression data of 11,756 cells from atherosclerotic coronary arteries in the Gene Expression Omnibus (GEO) database and conducted a comprehensive analysis. After quality control, 11,722 cells were obtained for subsequent analysis. Next, we performed t-distributed stochastic neighbor embedding (t-SNE) to reduce the dimensions of cells (Figure 1A and S1A). We manually annotated and classified 20 major clusters based on known marker genes (Figure S1B).

To investigate the expression pattern of m^6^A regulators in atherosclerotic cell types, we displayed the expression distribution of several m^6^A regulators across major cell types, with most being widely distributed, suggesting that m^6^A regulators may play a general role in atherosclerosis (Figure 1B). We then comprehensively evaluated the difference of the average RNA expressions of m^6^A regulators in different cell types. We observed that the expression levels of m^6^A regulators differ among different cell types (Figure 1C). For example, *IGF2BP3* exhibited the highest expression level in macrophages. The expression profiles of 23 m^6^A regulators were visualized using t-SNE, depicting the expression level of each m^6^A regulator per cell (Figure 1D and S1C).

Furthermore, consensus clustering analysis based on all genes and m^6^A regulators expression presented that different cell types did not cluster according to the expressions of m^6^A regulators (Figure 1E). However, there was a clear clustering of cell types based on all genes expression, indicating that m^6^A regulators may have similar regulatory mechanisms in atherosclerotic cell types. To determine whether m^6^A regulators could function as marker genes in various cell types, we performed an intersection analysis between m^6^A regulators and marker genes in different cell types. The absence of overlap between the marker genes and m^6^A regulators indicated that m^6^A regulators did not have a potential regulatory effect on function as marker genes (Figure 1F).

The pathogenesis of atherosclerosis is accompanied by physiological and pathological changes in important cell types 2. To analyze the association between m^6^A regulators and key functions, we conducted correlation analysis to assess the potential relationships between m^6^A regulators and various biological functional terms of atherosclerosis (Figure S1D). We observed that m^6^A regulators' scores were significantly correlated with the scores of “Cell cell adhesion” and “Cell surface receptor signaling pathway involved in heart development” pathways. Notably, we revealed that the same m^6^A regulator may have similar or opposite regulatory effects across different cell types (Figure 1G). Thus, m^6^A regulators may play specific roles in regulating the function of diverse cell types in atherosclerosis.

To further explore the relationship of m^6^A regulators and pathway-related genes, we calculated the correlations between m^6^A regulators and representative genes within the cell surface receptor pathway for key cell types, focusing on genes associated with atherosclerosis in GeneCards 30. We observed that the same m^6^A regulator showed varying levels of correlation with genes across different cell types (Figure S1E). Overall, m^6^A regulators showed cell-type-specific associations with pathways and genes, underscoring the value of studying their role in atherosclerosis through single-cell analysis.

### ALKBH5 regulates EC proliferation and migration involved in sprouting angiogenesis

**Figure 2:**
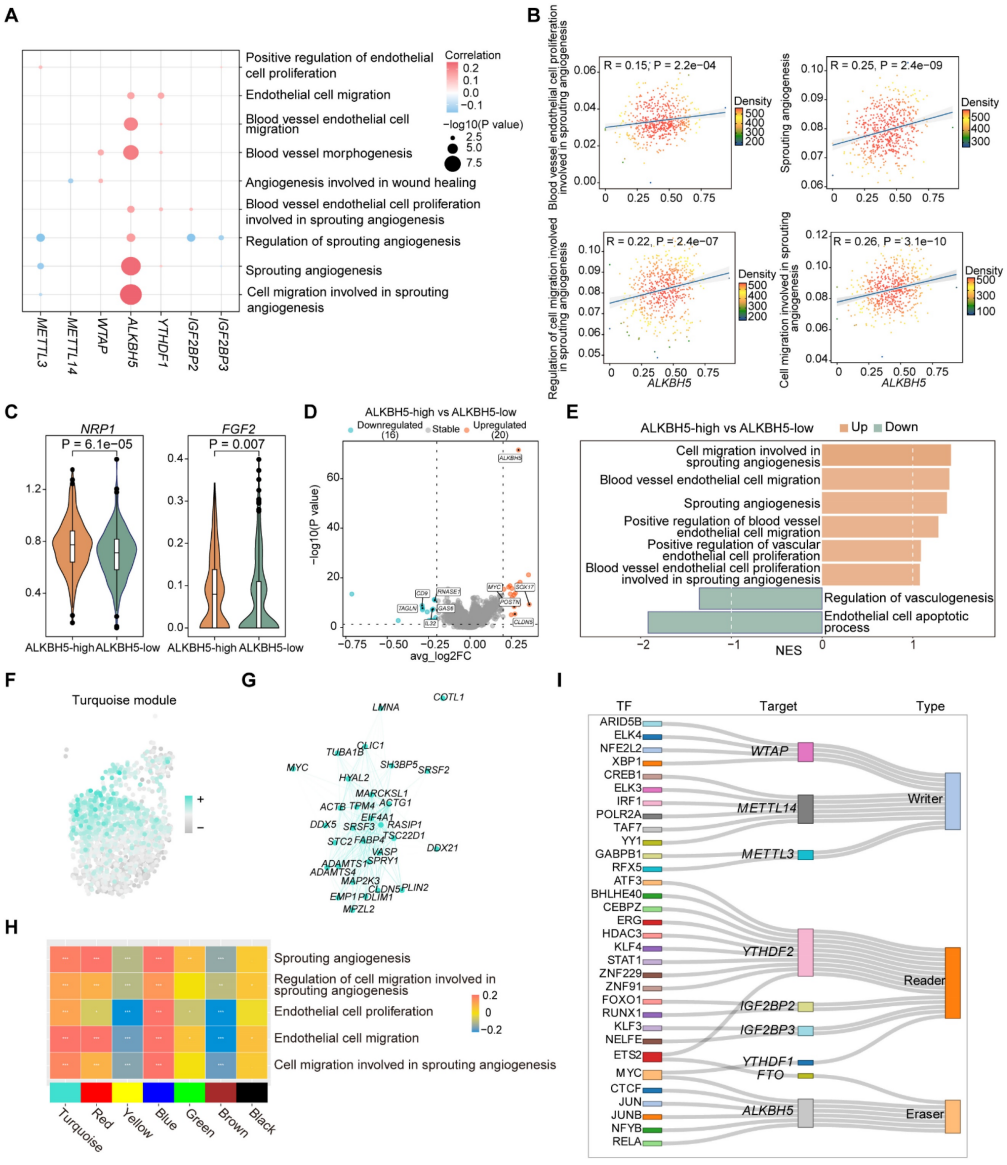
** ALKBH5 regulates EC proliferation and migration involved in the signaling pathway of sprouting angiogenesis.** (A) Dot plot showing the correlation between signatures of disease-related pathways and the expression levels of key m^6^A regulators in ECs. *P* value was calculated by Pearson correlation. (B) Correlations between the expression of *ALKBH5* and signatures of EC-related functional pathways. (C) Violin plots showing the expression levels of *NRP1* and *FGF2* in ALKBH5-high group and ALKBH5-low group. The statistical differences between the groups were determined through Wilcoxon rank test. (D) Volcano plot showing DEGs between ALKBH5-high group and ALKBH5-low group. Significant DEGs were shown in orange (upregulation) or cyan (downregulation). (E) Bar chart showing the results of GSEA. NES, normalized enrichment score. Brown represents upregulated pathways and green represents downregulated pathways. (F) Module feature plot showing the distribution of turquoise module. Sorted based on the values of hMEs, with the color depth of the points indicating the level of hMEs values. (G) *ALKBH5* co-expressed genes network showing the top 30 hub genes within the turquoise module. Each node in the graph represents a gene, and each edge represents a co-expression relationship, with the opacity of the edges scaled according to the strength of the co-expression relationship. (H) The correlation between signatures of pathways related to ECs proliferation and migration and gene modules. (I) Sankey diagram showing TFs regulating the expression levels of m^6^A regulators.

To elucidate the potential regulatory role of m^6^A regulators in ECs, we analyzed the correlation between m^6^A regulators expressions and key pathways. We observed that *ALKBH5* exhibited significant positive correlation with pathways in ECs (Figure 2A), which was associated with EC proliferation and migration involved in sprouting angiogenesis (Figure 2B). These functions are closely associated with the development of the disease, which may potentially lead to the disruption of plaques and the occurrence of inflammatory reactions 31. We further focused on the genes in these pathways, and we found that *ALKBH5* showed a significant positive correlation with these genes (Figure S2A). To further investigate the role of ALKBH5 in ECs, we divided ECs into two groups (ALKBH5-high group and ALKBH5-low group) based on *ALKBH5* expression. NRP1 is a cell-surface receptor known to be involved in cardiovascular development and angiogenesis. FGF2 is a growth factor that stimulates pro-angiogenic behavior in endothelial progenitor cells, resulting in increased proliferation, migration and tube formation capacity 32. By comparing the expression levels of pathway genes between two groups, we found that several genes (*NRP1*, *FGF2*, *PLK2*, *EGR3*, *KLF4*, *KLF2*) were highly expressed in ALKBH5-high group (Figure 2C and S2B).

Next, to compare the differential genes and functions between ALKBH5-high and low groups, we performed differential expression analysis (Figure 2D). Gene Ontology (GO) analysis based on differentially expressed genes (DEGs) revealed pathways enriched in ALKBH5-high group (Figure S2C). Additionally, gene set enrichment analysis (GSEA) revealed that pathways related to cell migration and proliferation during sprouting angiogenesis process were more active in ALKBH5-high group (Figure 2E).

In addition, weighted correlation network analysis (WGCNA) can be used to identify highly correlated gene modules and assist in the identification of key genes 33. High dimensional weighted gene co-expression network analysis (hdWGCNA) was employed to identify gene module co-expressed with *ALKBH5* 34. We identified seven gene modules with distinct functional characteristics (Figure S2D-S2E), among which we found several co-expressed genes with *ALKBH5* in the turquoise module (Figure 2F-2G). Functional enrichment analysis of the genes in the turquoise module revealed significant pathways related to angiogenesis and cell migration (Figure S2F). Subsequent correlation analysis between these functional pathways and the gene modules demonstrated a strong association with the turquoise module (Figure 2H).

In order to predict TFs that regulate *ALKBH5* expression, we performed single-cell regulatory network inference and clustering (SCENIC), which revealed that *ALKBH5* was regulated by multiple TFs (Figure 2I). CTCF is a chromatin-binding factor that can bind to DNA at specific sequence and plays a crucial role in the regulation of epigenetic modifications. MYC is a broadly acting TF that can bind to VEGFA promoter, thereby promoting VEGFA production and sprouting angiogenesis 35. JUN is a member of the AP-1 family and can be involved in the regulation of many biological processes, including cell proliferation, differentiation, apoptosis and inflammation. These findings indicated that CTCF, MYC and JUN may regulate *ALKBH5*, influencing cell proliferation and migration in sprouting angiogenesis.

### ALKBH5 has the potential role in activating ECs phenotypic changes and promoting ECs intercellular communication

**Figure 3:**
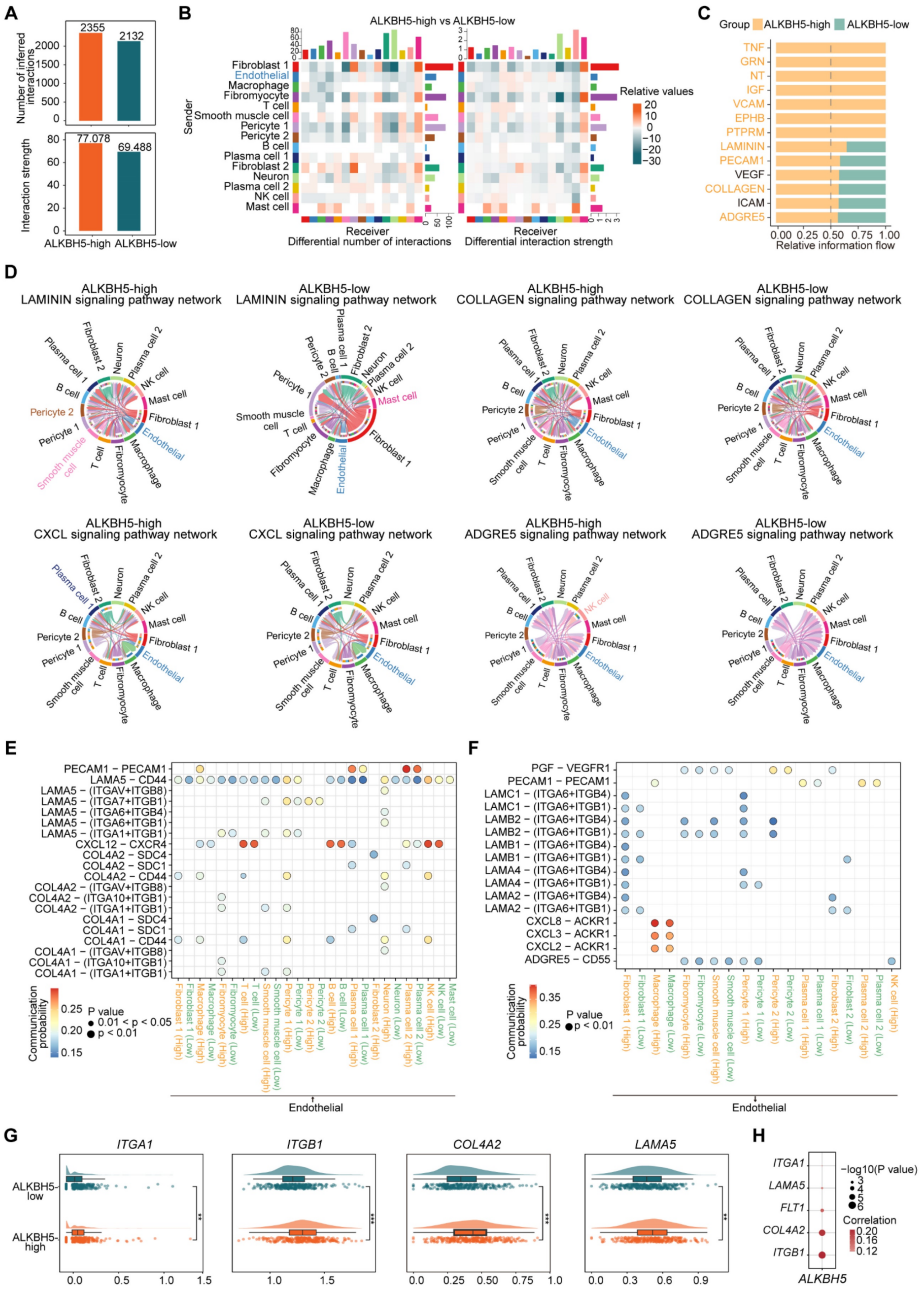
** ALKBH5 regulates ligand-receptor pairs associated with ECs phenotypic activation.** (A) The total number of interactions and the interaction intensity of the inferred cell-cell communication networks in ALKBH5-high and ALKBH5-low groups. (B) Heatmaps showing the differences in the number or intensity of interactions between all cell types in ALKBH5-high group and ALKBH5-low group. Orange represents an increase in ALKBH5-low group compared to ALKBH5-high group, while green represents a decrease. The colored bar graph at the top represents the total value of each column displayed in the heatmap (incoming signal). The colored bar graph on the right represents the total value of each row (outgoing signal). (C) Comparison of several signaling pathways in ALKBH5-high and ALKBH5-low groups. (D) Chord plots showing the signaling pathways (LAMININ, COLLAGEN, CXCL, ADGRE5) of aggregated cell-cell communication networks at the signaling pathway level in ALKBH5-high and ALKBH5-low groups. Cell types with altered communication were colored. (E) Comparison of specific ligand-receptor pairs from ECs to other cell types between ALKBH5-high group and ALKBH5-low group. (F) Comparison of specific ligand-receptor pairs from other cell types to ECs between ALKBH5-high group and ALKBH5-low group. (G) Violin plots showing the expression levels of ligand and receptor genes in ALKBH5-high group and ALKBH5-low group. The statistical differences between the groups were determined through Wilcoxon rank test. (H) Correlations between the expression levels of *ALKBH5* and ligand-receptor genes. *P* value was calculated by Pearson correlation.

The vascular endothelium, which forms a continuous cellular lining layer within the cardiovascular system, is a crucial site for key regulatory elements within this homeostatic framework 36. To explore ALKBH5's impact on ECs cellular communication, we compared networks between ALKBH5-high and ALKBH5-low groups (Figure 3A-3B). We analyzed several signaling pathways as well as the signaling pathways when ECs act as ligand cells (Figure 3C and S3A). It has been reported that the formation of plaques tends to occur at vessel bifurcations, where ECs are exposed to disturbed laminar flow, leading to endothelial phenotype activation 37. Endothelial activation involves the upregulation of pro-inflammatory cytokines and chemokines, as well as increasing expression of adhesion molecules, which are critical for recruiting immune cells 38. We focused on several highly active pathways significantly associated with the activation of the endothelial phenotype (LAMININ, CXCL, ADGRE5, PECAM1) in ALKBH5-high group, in addition to those related to extracellular matrix (COLLAGEN) and angiogenesis (VEGF) (Figure 3D and S3B). The pathways involved in cell adhesion and angiogenesis of ECs were positively correlated with *ALKBH5* (Figure S3C).

Furthermore, our study demonstrated changes in the communication of potential ligand-receptor pairs in two groups. It was observed that ECs primarily interacted with immune cells through COL4A1-CD44 and COL4A2-CD44 ligand-receptor pairs (Figure 3E). In atherosclerosis, ECs communicated extensively with pericyte cells and SMCs through LAMININ (LAMA5-(ITGA1+ITGB1), LAMA5-(ITGA7+ITGB1), LAMB2-(ITGA6+ITGB4), LAMB2-(ITGA6+ITGB1)) and COLLAGEN (COL4A1-(ITGA1+ITGB1), COL4A2-(ITGA1+ITGB1)) pathways (Figure 3E-3F). We identified the molecules or genes associated with the upregulated signaling pathways in ALKBH5-high group (Figure S3D). Additionally, the expression levels of these ligand and receptor genes were significantly elevated in ALKBH5-high group compared to ALKBH5-low group, and *ALKBH5* exhibited a significant positive correlation with these genes (Figure 3G-3H and S3E). Overall, ALKBH5 may play a regulatory role in activating EC phenotype, worsening disease progression by promoting angiogenesis, cell migration and adhesion in atherosclerosis.

### *ALKBH5* upregulation in HCAECs drives ox-LDL-induced angiogenesis and is reversed by knockdown

**Figure 4:**
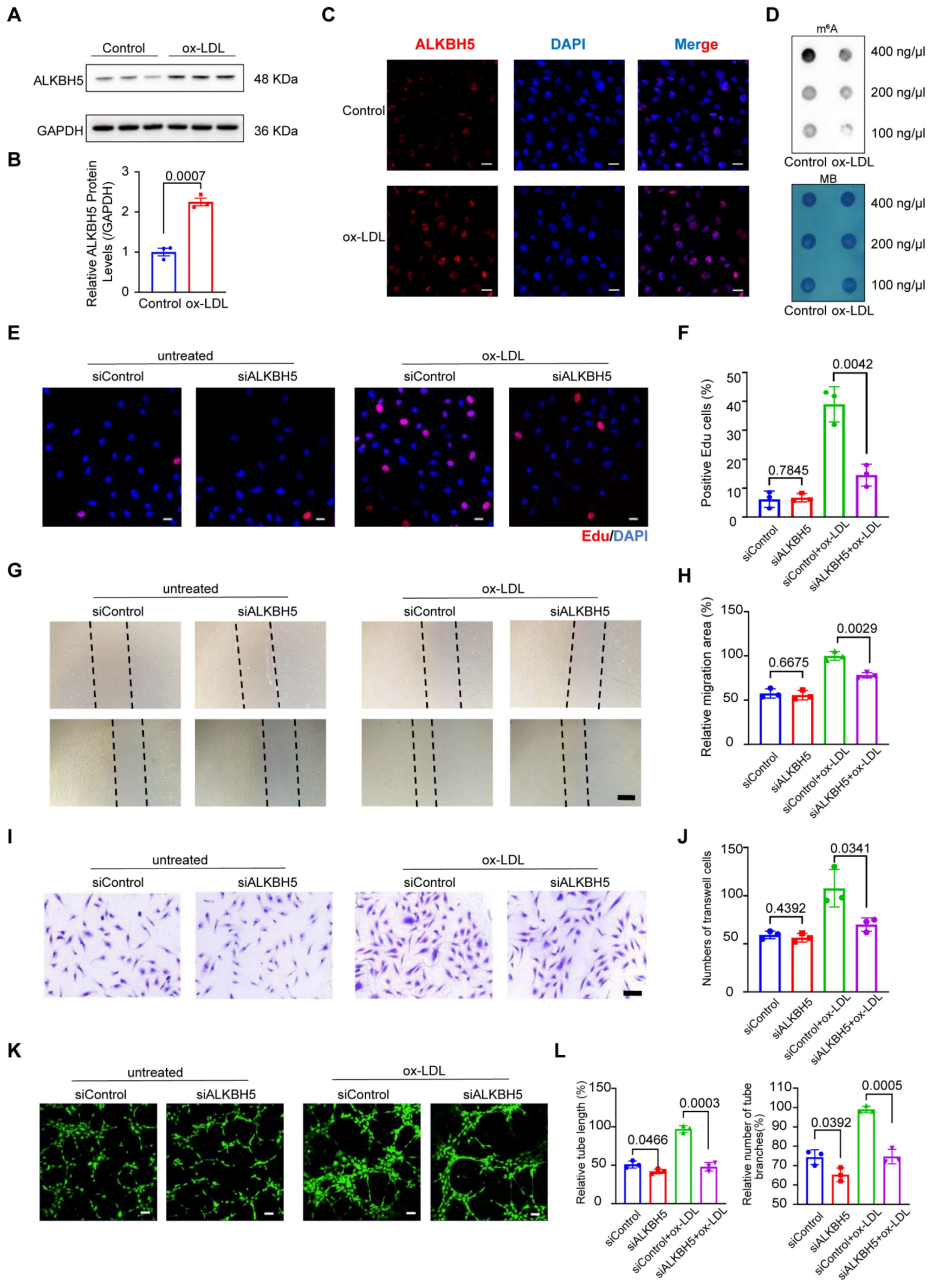
** The upregulation of ALKBH5 in HCAECs in response to ox-LDL stimulation promotes angiogenesis.** (A) Representative western blot images of ALKBH5 levels in control and HCAECs treated with 50 μg/mL ox-LDL at 48 hours. (B) Quantification of ALKBH5 levels in control and HCAECs treated with 50 μg/mL ox-LDL at 48 hours (n=3). (C) Representative immunofluorescence images to detect ALKBH5 expression in 50 μg/mL ox-LDL-induced HCAECs at 48 hours. Scale bars, 20 μm. (D) Dot blot assay using an anti-m^6^A antibody in 50 μg/mL ox-LDL-induced HCAECs. MB staining was included as a loading control. Total RNA concentration: 400 ng/μL, 200 ng/μL, 100 ng/μL. (E) Cell proliferation was measured by EdU staining. (F) The percentage of EdU-positive HCAECs (red) was quantified (n=3). Scale bars, 20 μm. (G) HCAECs migration ability was measured by the Scractch Closure assay. (H) Quantifications of relative migration area of HCAECs were made (n=3). Scale bars, 500 μm. (I) HCAECs migration ability was measured by the Transwell assay. (J) Quantifications of migration cell number of HCAECs were made (n=3). Scale bars, 100 μm. (K) Images of the tube formation assay. (L) Quantitative analysis of the tube length and branches number (n=3). Scale bars, 100 μm. Data are presented as mean±SD. T test followed by a Normality test was used.

To explore whether *ALKBH5* expression changes in atherosclerotic ECs, human coronary artery endothelial cells (HCAECs) were stimulated by oxidized low-density lipoprotein (ox-LDL) and interleukin (IL)-1β, which are key factors in atherosclerotic angiogenesis. In our initial experiments, *ALKBH5* expression increased significantly in treated HCAECs after exposure to ox-LDL (50 μg/mL) and IL-1β (10 ng/mL) (Figure 4A-4C and S4A-S4B). Besides, dot blot assays revealed that m^6^A methylation levels decreased in both treatments (Figure 4D and S4C). Our results showed that ox-LDL and IL-1β stimulation upregulated *ALKBH5* in HCAECs, suggesting a role for this factor in regulating ECs function and angiogenesis in atherosclerosis.

To further investigate the role of ALKBH5 in atherosclerotic angiogenesis, we knocked down its expression in HCAECs using small interfering RNA (siRNA) and compared cell proliferation and migration abilities of HCAECs. As expected, *ALKBH5* knockdown increased m^6^A methylation (Figure S4D-S4F). Furthermore, we noticed that EdU assays showed higher proliferation and viability in ox-LDL-induced HCAECs, yet *ALKBH5* knockdown reversed these effects (Figure 4E-4F). Scratch and Transwell assays indicated that ox-LDL increased cell migration, which could be decreased by *ALKBH5* knockdown (Figure 4G-4J). Additionally, *ALKBH5* knockdown was found to diminish the healing and migration capabilities of HCAECs (Figure 4K-4L). Bioinformatics analysis revealed that ALKBH5 influenced the expressions of genes (*NRP1*, *FGF2*, *PLK2*, *EGR3*, *KLF4*, *KLF2*), and to explore this impact, we conducted western blot assays and found that ox-LDL increased *KLF2* and *PLK2* expression in HCAECs, and *ALKBH5* knockdown reduced this effect (Figure S4G-S4H). To investigate whether m^6^A modification is involved in the regulation of the above target genes, we used the SRAMP m^6^A modification site prediction tool to predict the m^6^A sites in these target genes and found that the above target genes were rich in m^6^A modifications. Then, we designed primers for m^6^A modification sites in target genes and conducted MeRIP-qPCR to verify the m^6^A modification. The m^6^A modification levels of *KLF2* and *PLK2* in ox-LDL-induced HCAECs were higher in siALKBH5-transfected cells than in siControl-transfected cells (Figure S4I), suggesting that their m^6^A were regulated by ALKBH5. To exclude the influence of ALKBH5 on the expression of its upstream TFs (CTCF, MYC, JUN), we conducted western blot assays and found that *ALKBH5* knockdown did not affect CTCF, MYC and JUN expressions in ox-LDL-induced HCAECs (Figure S4J-S4K), suggesting that these TFs regulate the expression of *ALKBH5*.

### Upregulation of *WTAP* expression increases plaque instability by promoting cell proliferation and migration in SMCs

**Figure 5:**
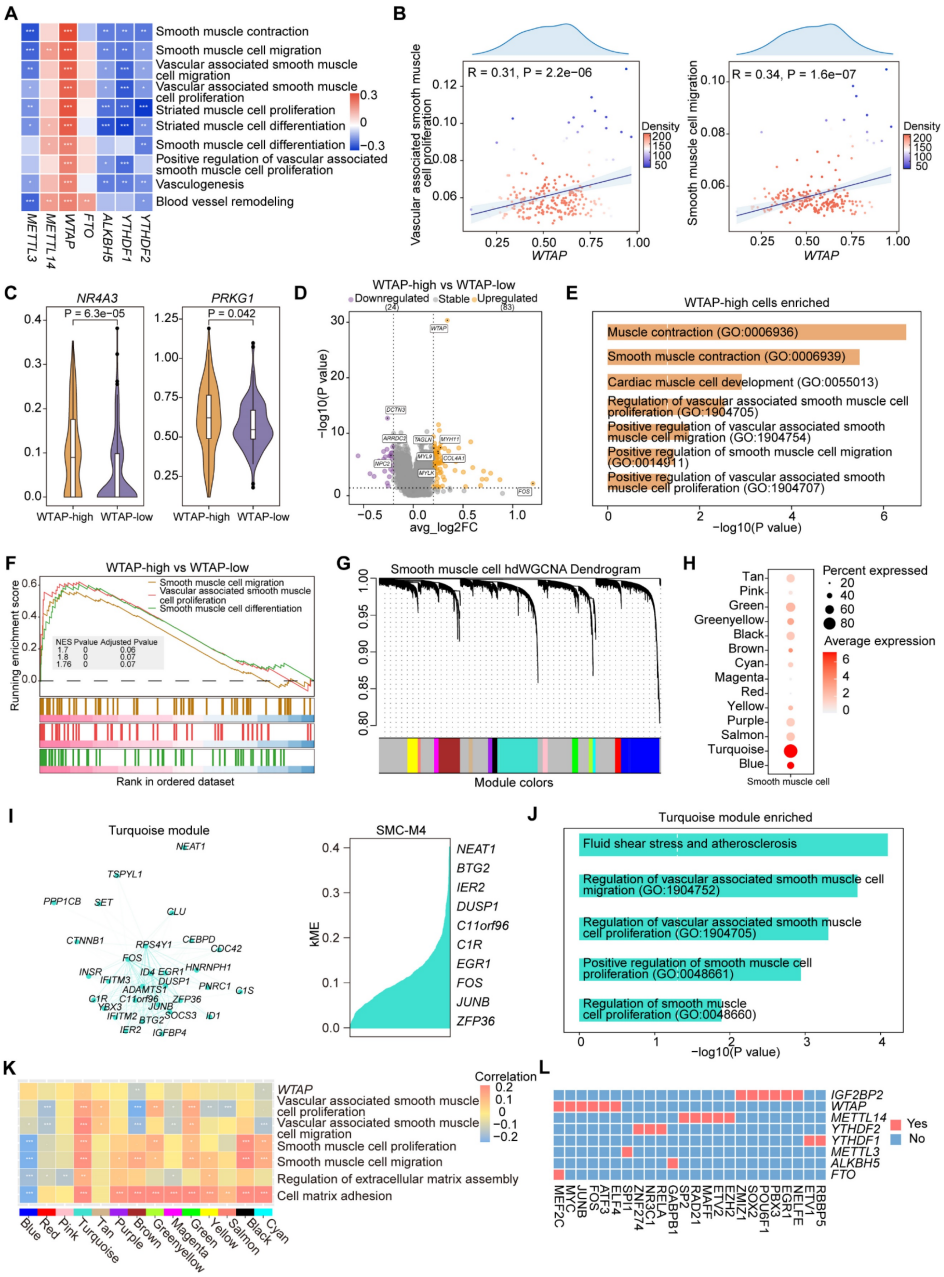
** WTAP specifically regulates the proliferation and migration of SMCs.** (A) Correlation analysis between the expression levels of selected m^6^A regulators and signatures of disease-related pathways in SMCs. *P* value was calculated by Pearson correlation. (B) Correlations of *WTAP* expression with signatures of “Vascular associated smooth muscle proliferation” and “Smooth muscle cell migration” pathways. *P* value was calculated by Pearson correlation. (C) Violin plots showing the expression levels of *NR4A3* and *PRKG1* in WTAP-high group and WTAP-low group. The statistical differences between two groups were determined through Wilcoxon rank test. (D) Volcano plot showing DEGs in WTAP-high group and WTAP-low group. Significant DEGs were shown in orange (upregulation) and purple (downregulation). (E) Representative GO terms and pathways of upregulated genes in WTAP-high group. (F) GSEA showing three SMC-related pathways. (G) Gene modules detected through hdWGCNA in atherosclerotic SMCs. (H) Overall expression levels of hMEs in different modules within SMCs. (I) *WTAP* co-expressed genes network showing the top 30 hub genes (left, network) and top 10 hub genes ranked by kME (right) within the turquoise module. In the network, each node represents a gene and each edge represents a co-expression relationship, with the opacity of the edges scaled according to the strength of the co-expression relationship. (J) Disease-related pathways enriched by 200 genes ranked by kME within the turquoise module. (K) Correlation of all gene modules with *WTAP* and signatures of SMC-related signaling pathways. (L) Heatmap showing the TFs that regulate m^6^A regulators in SMCs.

To investigate the regulatory role of m^6^A regulators in SMCs, we performed correlation analysis and found that *WTAP* was significantly positively correlated with several pathways, including pathways related to cell migration, proliferation and vasculogenesis (Figure 5A-5B). Notably, *WTAP* was significantly correlated with genes associated with SMC proliferation and migration (Figure S5A). We then divided the cells into two groups based on *WTAP* expression, which were WTAP-high group and WTAP-low group. *NR4A3* is involved in regulating proliferation, survival, differentiation, metabolism, and inflammation in various cell types. *PRKG1* is a serine and threonine protein kinase that can influence the expression of SMC contractile proteins. Our analysis showed that the expression of the genes associated with SMC proliferation and migration was higher in WTAP-high group (Figure 5C and S5B).

To compare the differential genes and functions between the two groups, we performed a differential expression analysis. DEGs between two groups were identified (Figure 5D). Then, we conducted GO analysis on DEGs, revealing several upregulated functional pathways likely affected by WTAP (Figure 5E). GSEA revealed that pathways associated with SMC proliferation and migration were more active in WTAP-high group (Figure 5F).

In addition, we conducted hdWGCNA to identify gene modules co-expressed with *WTAP*. We constructed co-expression networks that leading to the identification of 14 gene modules (Figure 5G and S5C). The harmonized module eigengenes (hMEs) within the turquoise module exhibited the highest overall expression levels within the SMCs (Figure 5H). We identified *WTAP* and its co-expressed genes in turquoise module (Figure 5I). GO analysis revealed that these genes were involved in pathways of SMC proliferation and migration (Figure 5J). Next, we found a significant positive correlation between the turquoise module and these pathways (Figure 5K). This finding further supported the role of *WTAP* and its co-expressed genes in the regulation of SMC proliferation and migration.

Finally, we performed SCENIC to identify TFs that could regulate *WTAP*. *WTAP* was regulated by MEF2C, FOS and ATF3 (Figure 5L), which have been reported to be involved in SMC proliferation and migration. The investigation conducted by Lu *et al.* observed that endothelial MEF2C in the internal elastic lamina inhibited SMC migration through fenestrations 39. Miao *et al.* demonstrated the fundamental role of the mtROS/c-Fos/LOX-1 pathway in promoting ox-LDL uptake and vascular smooth muscle cells (VSMCs) derived foam cells formation during atherosclerosis 40. Lv *et al.* found that overexpression of *ATF3* led to increased expression of *MMP* and enhanced migration of SMCs 41. The results suggested that important TFs (MEF2C, FOS, ATF3) regulated *WTAP*, making WTAP a crucial target in atherosclerotic SMCs with potential regulatory effects on their proliferation and migration.

### *WTAP* upregulation in HCASMCs drives ox-LDL-induced proliferation, migration, and this effect is reversed by its knockdown

To explore whether *WTAP* expression changes in atherosclerotic SMCs, we stimulated human coronary SMCs (HCASMCs) with ox-LDL. In our initial experiments, *WTAP* expression significantly increased in HCASMCs treated with ox-LDL (100 μg/mL) (Figure S6A-S6B). To further investigate the role of WTAP in driving ox-LDL-induced proliferation and migration, we knocked down its expression in HCASMCs using siRNA and compared the cell proliferation and migration abilities of these cells. As expected, EdU assays showed higher proliferation and viability in ox-LDL-induced HCASMCs, yet *WTAP* knockdown reversed these effects (Figure S6C-S6D). Scratch assays indicated that ox-LDL increased migration, which could be decreased by *WTAP* knockdown (Figure S6E-S6F). Single-cell data analysis revealed that WTAP affects the expression level of *PRKG1*. Through experimental verification, we found that knocking down *WTAP* in HCASMCs led to a decrease in the expression of *PRKG1* (Figure S6G-S6H). We then conducted MeRIP-qPCR to verify that m^6^A modification is involved in the regulation of *PRKG1* gene expression. The m^6^A modification level of *PRKG1* in ox-LDL-induced HCASMCs was less in siWTAP-transfected cells than in siControl-transfected cells (Figure S6I). SCENIC results indicated that TFs (FOS, MEF2C) regulate *WTAP*. Experimental verification demonstrated that the knockdown of *WTAP* did not impact their expression (Figure S6J-S6K), evidencing that FOS and MEF2C are regulators of *WTAP*.

### WTAP is an important regulator in SMC phenotypic transformation

**Figure 6:**
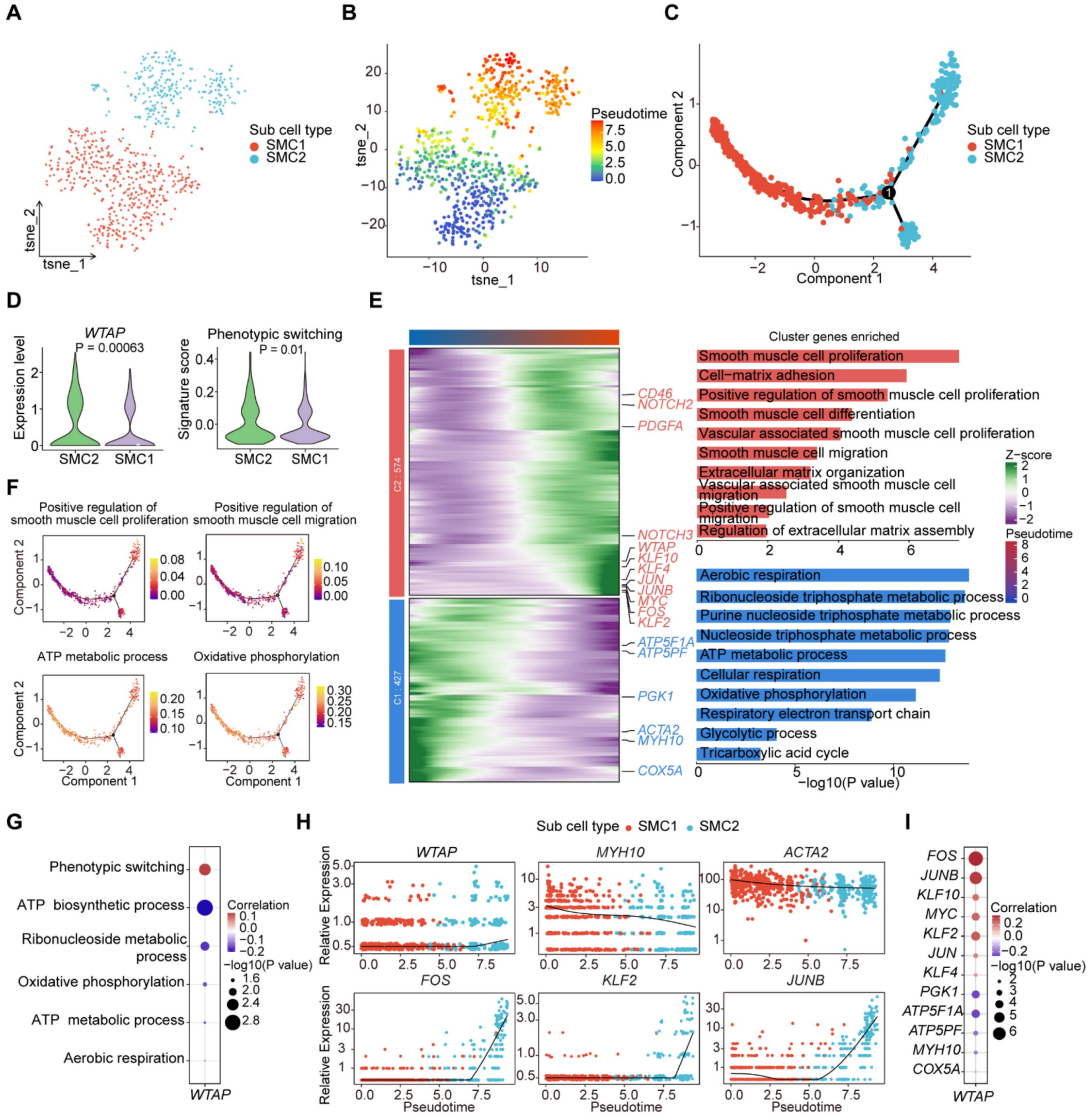
** WTAP regulates the phenotypic transformation of SMCs.** (A) T-SNE plot showing the subclusters of SMCs. (B) Projection of the pseudotime trajectory onto the t-SNE plot. (C) The order of SMC1 and SMC2 along the pseudotime axis in the 2D state space defined by Monocle2. The orders of the cells were inferred from the expression of the top 100 DEGs using “FindAllMarkers” function, with each point corresponding to a single cell. (D) The expression level of *WTAP* and signature score of “Phenotypic switching” pathway. The statistical differences between two groups were determined through t-test. (E) Heatmap showing gene expression profiles alongside the pseudotime of SMC1 and SMC2, which were divided into two clusters based on their expression patterns (left). The enriched GO pathways corresponding to the heatmap clustering were shown on the right. Blue represents cluster 1 and red represents cluster 2. (F) AUCell scores of disease-related pathways enriched along the evolutionary trajectory of SMCs. (G) Correlations between the expression level of *WTAP* and signatures of disease-related pathways in SMCs. (H) Dynamic alterations in *WTAP* and other key genes during phenotypic transformation. (I) Correlations between the expression levels of *WTAP* and phenotypic-related genes, as well as metabolism-related genes in SMCs.

In atherosclerosis, SMCs undergo phenotypic transformation characterized by an increase in cell proliferation, migration, and extracellular matrix synthesis, as well as a decrease in the expression of contractile markers 42,43. To investigate whether WTAP modulates the process of phenotypic transformation of SMCs, we re-clustered SMCs and obtained two SMC subclusters: SMC1 and SMC2 (Figure 6A). Pseudotime trajectory analysis revealed potential transformation trajectories from SMC1 to SMC2 (Figure 6B-6C). *WTAP* expression and signature score of “Phenotypic switching” pathway were more pronounced in SMC2 (Figure 6D). To better compare the pseudotime trajectories between two subclusters, we visualized the dynamic changes of genes through heatmaps and conducted GO enrichment analysis (Figure 6E). The results suggest that SMC2 may represent a subcluster of the synthetic phenotype that undergoes cell proliferation and migration processes. In addition, we showed representative pathways during the pseudotime trajectory (Figure 6F and S7A). The representative functional pathways of two subclusters had opposite correlations with *WTAP* (Figure 6G), suggesting that the phenotype of SMCs changed along the pseudotime trajectory.

Moreover, we demonstrated that genes related to functions showed distinct expression patterns along the pseudotime trajectory (Figure S7B), and these genes related to SMC phenotype were summarized with reference to Mao *et al.* 44. Notably, representative energy-related genes (such as *ATP5PF*, *NDUFB2*) displayed higher expression levels in the early stages, while those associated with the proliferative and migratory phenotype (such as *JUN*, *FOS*) exhibited increased expression in the later stages (Figure 6H and S7C-S7D). In addition,* KLF4* has been shown to induce phenotypic change in cultured VSMCs in response to platelet-derived growth factor, phospholipids, or IL-1β 42. Conditional knockout of *KLF4* in VSMCs could significantly reduce the size of plaques, increasing the area of the fibrous cap and improving the stability of plaques 45. We observed that *KLF4* expression gradually increased during pseudotime trajectory, while *ACTA2* gradually decreased (Figure S7B). *WTAP* was negatively correlated with genes associated with energy metabolism and positively correlated with genes related to phenotypic transformation (Figure 6I). Overall, WTAP contributes to the phenotypic transformation of SMCs in atherosclerosis.

### METTL3 and YTHDF2 regulate macrophage activation and differentiation thereby promoting atherosclerosis

**Figure 7:**
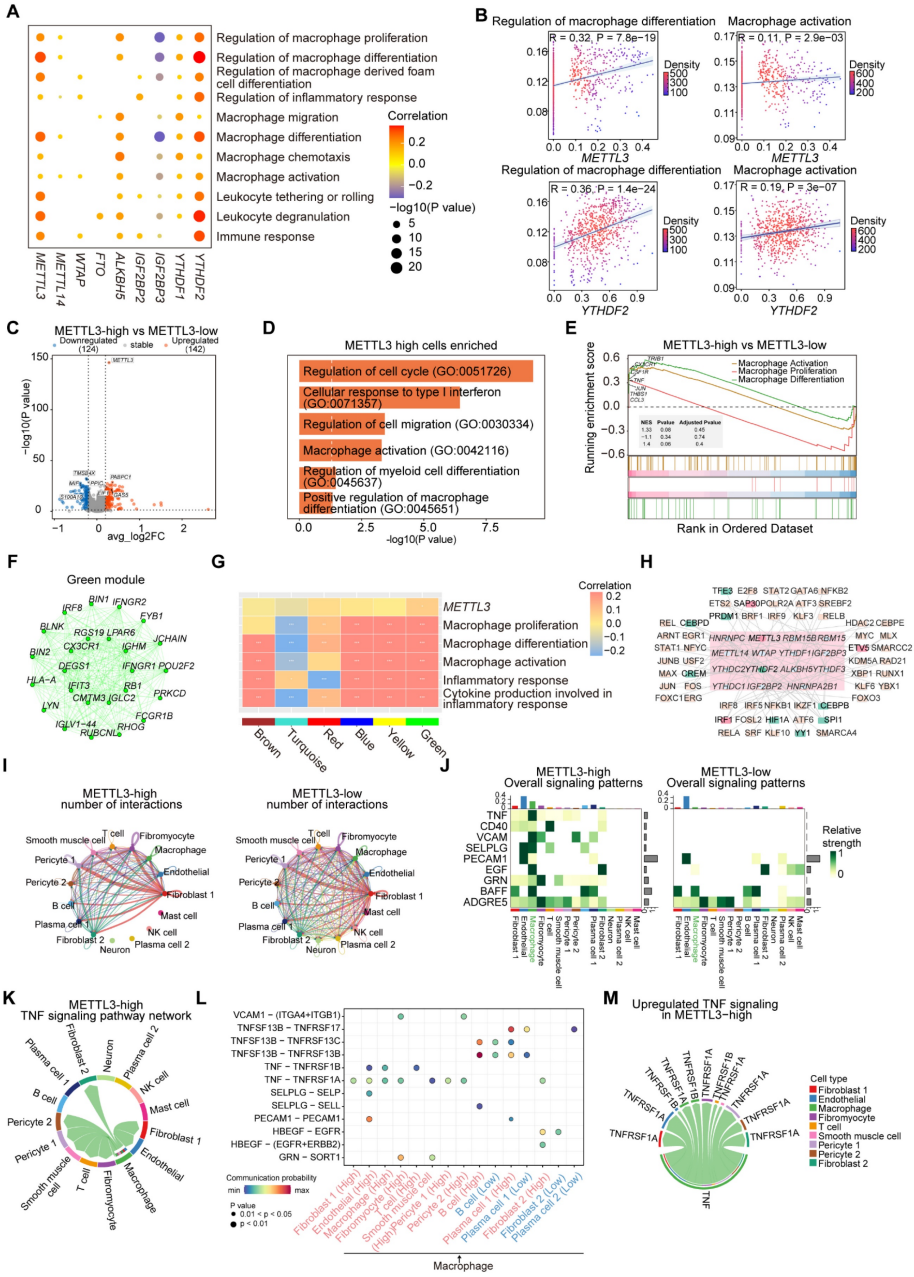
**METTL3 regulates the activation, differentiation and the intercellular communication of macrophages.** (A) Dot plot showing the correlation between signatures of disease-related pathways and the expression levels of key m^6^A regulators in macrophages. *P* value was calculated by Pearson correlation. (B) Correlations between the expression of *METTL3* and *YTHDF2* with signatures of macrophage-related functional pathways. (C) Volcano plot showing DEGs between METTL3-high group and METTL3-low group. Significant DEGs are shown in orange (upregulation) or blue (downregulation). (D) Representative GO terms and pathways of upregulated genes in METTL3-high group. (E) GSEA showing three macrophage-related pathways. (F) *METTL3* co-expressed genes network showing the top 25 hub genes within the green module. Each node in the graph represents a gene, and each edge represents a co-expression relationship, with the opacity of the edges scaled according to the strength of the co-expression relationship. (G) The correlation between gene modules and *METTL3*, as well as the signaling pathways related to macrophages. (H) Network plot showing TFs regulating m^6^A regulators. m^6^A regulators are located in the central pink box surrounded by TFs. TFs regulating *METTL3* expression are colored pink, and those for *YTHDF2* expression are green. (I) Comparison of the number of interactions between all cell types in METTL3-high and METTL3-low groups. (J) Comparison of several signaling pathways in METTL3-high and METTL3-low groups. (K) Chord plot showing TNF signaling pathway of cell-cell communication network present only in METTL3-high group. (L) Comparison of specific ligand-receptor pairs from macrophages to other cell types between METTL3-high group and METTL3-low group. (M) Chord diagram showing the upregulated ligand-receptor pairs of the TNF signaling pathway.

To investigate the role of m^6^A regulators in macrophages, we conducted correlation analysis between m^6^A regulators and macrophage-related functional pathways. The results indicated that *METTL3* and *YTHDF2* were significantly correlated with pathways involved in macrophage differentiation, proliferation, activation and chemotaxis (Figure 7A-7B). We created groups according to *METTL3* and *YTHDF2* expression levels respectively: METTL3-high and METTL3-low groups, YTHDF2-high and YTHDF2-low groups. Further, we discovered that the expressions of several important genes including *TRIB1*, *RB1*, *IFNGR1* and *CX3CR1*, were higher in METTL3-high and YTHDF2-high groups (Figure S8A). CX3CR1, a transmembrane protein and chemokine, mediates the recruitment of macrophages and monocytes to inflammatory plaques. It has been reported that ginsenoside RB1 enhances the stability of plaques by inducing autophagy in macrophages 46, thereby reducing lipid accumulation in macrophage-derived foam cells. TRIB1-mediated conservative mechanisms may promote foam cells formation in atherosclerotic plaques 47.

To explore the differences in genes and functions between METTL3-high group and METTL3-low group, as well as YTHDF2-high group and YTHDF2-low group, we performed differential expression analysis. We found more DEGs upregulated in METTL3-high and YTHDF2-high groups (Figure 7C and S8B). Functional enrichment analysis revealed that pathways related to macrophage differentiation and activation were significantly enriched in METTL3-high group (Figure 7D). In YTHDF2-high group, “Macrophage differentiation” pathway was enriched (Figure S8C). We then further observed the activation of related pathways in METTL3-high and YTHDF2-high groups by GSEA (Figure 7E and S8D).

Furthermore, we identified the co-expressed genes of *METTL3* in green module using hdWGCNA (Figure 7F). Next, we conducted functional enrichment on the top 100 genes in the green module, noticing pathways associated with inflammatory response and cytokines (Figure S8E). To investigate the relationship between gene modules and various functions, we computed the correlation between all modules and related functional pathways by scoring. We found a significant positive correlation between the green module and *METTL3*, as well as functional pathways (Figure 7G).

Finally, we conducted SCENIC to predict TFs that regulate *METTL3* and *YTHDF2* in macrophages. We predicted multiple TFs regulating *METTL3* and *YTHDF2* (Figure 7H). In particular, IRF1 can activate genes involved in innate and acquired immune responses as a TF and tumor suppressor. The protein encoded by IRF1 has been implicated in cell proliferation, apoptosis and immune response, suggesting a potential regulatory role for TFs like IRF1 in *METTL3* expression. YY1 depletion has been shown to reduce cellular inflammatory response, as well as cholesterol homeostasis imbalance and lipid accumulation induced by ox-LDL. CEBPD promotes the accumulation of lipids in M1 macrophages, while CEBPB is crucial for the induction of gene expression in activated macrophages. Overall, YY1, CEBPD and CEBPB may affect macrophages activation and differentiation through regulating *YTHDF2*, thereby promoting inflammation and exacerbating the disease.

### METTL3 and YTHDF2 remodel macrophages intercellular networks via regulating adhesive and inflammatory function

To explore the effect of METTL3 on macrophages communication networks, we compared the overall number of cellular communications in METTL3-high and METTL3-low groups (Figure 7I). The METTL3-high group showed relatively less communication between macrophages and several other cell types (Figure S9A). Furthermore, we analyzed pathways in two groups and noted that certain pathways were more active in METTL3-high group (Figure 7J-7K and S9B). For example, TNF, CD40, SELPLG and PECAM1, which are associated with intercellular adhesion and the secretion of inflammatory factors, exhibited higher scores in METTL3-high group and were positively correlated with *METTL3* (Figure S9C).

To further investigate the potential impact of *METTL3* expression level on macrophages communication network, we compared the potential receptor-ligand pairs involved in signal flows between two groups (Figure 7J, 7L and S9D). We observed that the interactions between macrophages and various cell types were extensive, mediated particularly by TNF-TNFRSF1B and TNF-TNFRSF1A. In addition, PECAM1-PECAM1 and SELPLG-SELP were identified as the most significant contributors to the communication between macrophages and ECs. This interaction was intricately linked to the secretion of adhesive and chemotactic factors by ECs, which played a pivotal role in the recruitment of macrophages at the site of vascular inflammation or injury. We observed that T cells communicated with macrophages through CD40LG-(ITGAM+ITGB2), which may be associated with the activation of macrophages. We identified the molecules or genes associated with upregulated signaling pathways (TNF, CD40, SELPLG) in METTL3-high group (Figure 7M and S9E). Moreover, *METTL3* exhibited significant positive correlation with these ligand and receptor genes, and the expressions of genes (*SELPLG*, *ITGB2*) were significantly elevated in METTL3-high group (Figure S9F-S9G). Overall, METTL3 may facilitate macrophages interacting with other cells through increased adhesion and inflammation.

Likewise, we compared the number and strength of overall cell-cell interactions in YTHDF2-high and YTHDF2-low groups (Figure S10A). In YTHDF2-high group, macrophages exhibited heightened signaling intensity (Figure S10B). We found that highly active pathways including CXCL and ITGB2 were present in YTHDF2-high group. And certain inflammatory and adhesion-related signaling pathways (TNF, GRN, NECTIN, ALCAM, CD6, VCAM) appeared to be selectively active in macrophages in YTHDF2-high group (Figure S10C-S10D). Additionally, the scoring of these pathways showed that they had higher scores in YTHDF2-high group (Figure S10E). We highlighted the ligand-receptor pairs in these pathways (Figure S10F-S10G). The CXCL signaling pathway played a crucial role in mediating the interactions between macrophages and ECs, implicating a significant regulatory influence of YTHDF2 on the secretion of inflammatory factors. We identified the molecules or genes associated with upregulated signaling pathways (ITGB2, TNF, VCAM) in YTHDF2-high group (Figure S10H). Additionally, *YTHDF2* exhibited significant positive correlation with these ligand and receptor genes, which were significantly elevated in YTHDF2-high group (Figure S10I-S10J). Overall, YTHDF2 could promote macrophages to express specific inflammatory and adhesive genes.

Considering the pivotal role of macrophage-mediated inflammatory responses in atherosclerosis, we assessed the expression levels of *METTL3* in ox-LDL-induced macrophages (Figure S11A-S11B). Our findings revealed that deficiency of METTL3 in macrophages significantly reduced the mRNA expression of key pro-inflammatory cytokines, including IL-1β, IL-6, TNF-α, and inducible nitric oxide synthase (iNOS) (Figure S11C). Single-cell data analysis revealed that METTL3 regulates the expression level of *CX3CR1*. Through experimental verification, we found that knocking down *METTL3* in macrophages leads to a decrease in the expression of *CX3CR1* (Figure S11D-S11E). We conducted MeRIP-qPCR to verify that m^6^A modification is involved in the regulation of *CX3CR1* expression, which was prescribed as above. The m^6^A modification level of *CX3CR1* in ox-LDL-induced macrophages was less in siMETTL3-transfected cells than in siControl-transfected cells (Figure S11F). This further supports the regulatory role of METTL3 in inflammatory-related genes and its potential as a therapeutic target for atherosclerosis. SCENIC results indicated that IRF1 regulates the expression of *METTL3*, while experimental verification demonstrated that knockdown of *METTL3* did not impact the expression of *IRF1* (Figure S11G-S11H), thus evidencing that IRF1 is a regulator of METTL3.

## Discussion

Atherosclerosis is a chronic vascular disease involving functional abnormalities of multiple cell types. In this study, we comprehensively examined m^6^A modification in key cell types (ECs, SMCs, macrophages), revealing a cell-type-specific regulatory mechanism, with m^6^A regulators expressed widely and linked to multiple pathways. Furthermore, we focused on four crucial m^6^A regulators (ALKBH5, WTAP, METTL3, YTHDF2), not only demonstrating their cell-type-specific regulatory roles, but also identifying TFs that could regulate their expressions. Our research revealed the critical regulatory roles of m^6^A modification in intercellular communications during atherosclerotic progression, suggesting that m^6^A regulators could potentially impact atherosclerotic pathogenesis and mechanisms. Additionally, a series of experiments have validated the conclusions drawn from the single-cell data analysis. The experimental results demonstrated the roles of ALKBH5, WTAP and METTL3 in atherosclerotic processes, which are consistent with recent studies on m^6^A in atherosclerosis 48. Overall, we comprehensively investigated the single-cell atlas of m^6^A modification regulating cell-type-specific functions in atherosclerosis.

It has been reported that the pathogenesis of atherosclerosis is closely associated with cell-type-specific epigenetic modifications 20,49. Endothelial dysfunction is the earliest change detected in atherosclerotic pathogenesis, occurring in the vulnerable area of the arteries 50. Zhang *et al.* found METTL14 promotes miR-19a processing by pri-miR-19a methylation, enhancing EC proliferation and invasion 51. Depuydt *et al.* uncovered the pro-inflammatory role of intercellular signaling, in which ACKR1 derived from ECs interacts with inflammatory factors from myeloid cells and CSFR1 from myeloid cell subclusters interacts with CSF1 from various cell types 52. Similar to ECs, SMCs are thought to undergo phenotypic transformation, characterized by proliferation, dedifferentiation and migration to the site of intimal lesions 53. Chen *et al.* showed that METTL3-mediated m^6^A modification promotes VSMC phenotypic transformation and plaque development via miR-375-3p/PDK1 axis 54. Macrophages in atherosclerosis primarily derive from monocytes, which can further differentiate into foam cells and trigger inflammatory responses 55. Zhang *et al.* reported that METTL3 and YTHDF2 co-modifies PGC-1α mRNA, thereby promoting mitochondrial dysfunction and ox-LDL-induced monocyte inflammation 56. Chien *et al.* discovered that METTL3 plays a critical role in mediating the atherogenic inflammatory cascades 57. To date, numerous studies have explored the regulatory mechanisms of m^6^A regulators through bulk RNA-seq datasets or specific knockout experiments 27-28,58-59. However, these studies have limitations in uncovering cell-type-specific m^6^A modifications in atherosclerosis. Our research fills this gap by revealing the cell-type-specific functional roles of different m^6^A regulators in atherosclerosis at the single-cell level.

Our results indicated that *ALKBH5* upregulated in ECs induced cell proliferation and migration involved in sprouting angiogenesis. *ALKBH5* was upregulated in HCAECs in response to ox-LDL and IL-1β. Knockdown of *ALKBH5* not only increased m^6^A methylation level but also reversed the proliferative and migratory effects induced by ox-LDL. These findings are consistent with the hypothesis that ALKBH5 plays a critical role in atherosclerotic angiogenesis 48. We also confirmed that ALKBH5 influences the regulation of *KLF2* and *PLK2* by m^6^A methylation modification. Experiments demonstrated that *ALKBH5* is regulated by TFs (CTCF, JUN, MYC) and the expressions of TFs will not change after *ALKBH5* knockdown. Previous studies have discovered that m^6^A regulators could modulate ligand-receptor pairs in other diseases 60,61, yet few studies have explored their role in intercellular communication of atherosclerosis. We identified the potential effect of ALKBH5 on the ligand-receptor pairs during ECs phenotypic activation. In HCASMCs, the results indicated that *WTAP* was upregulated in response to ox-LDL, and its knockdown reversed the proliferative and migratory effects induced by ox-LDL. Previous studies have shown that VSMCs can be clustered into different subtypes, each characterized by a distinct cellular pseudotime trajectory 62,63. And SMCs exhibit substantial phenotypic plasticity in atherosclerosis 64. We also confirmed that WTAP influences the direct regulation of *PRKG1* by m^6^A methylation modification. One study has shown that PRKG1 affects the migration and proliferation of SMCs by regulating the recombination of smooth muscle cytoskeleton and activation of cell signaling pathways 65. In addition, m^6^A regulators (METTL3, YTHDF2) could promote macrophage activation and differentiation to exacerbate disease progression. Knockdown of *METTL3* reduced pro-inflammatory cytokines mRNA in macrophages, consistent with previous study highlighting the role of m^6^A methylation in immune regulation 66. Overall, we identified the specific functional roles of m^6^A regulators, which may offer novel therapeutic directions for the treatment of atherosclerosis.

While this study focuses on the role of m^6^A modification in atherosclerosis, other epigenetic modifications, namely DNA methylation and histone modifications, also play significant roles in atherosclerosis 67-69. For example, the hypomethylation of *COL15A1* leads to an increase in gene expression and an enhancement of the proliferative capacity of SMCs 69. HDAC3 (Histone deacetylase 3) is critical for endothelial survival and the development of atherosclerosis in response to disturbed flow 67. In addition, multiple epigenetic modifications can interact with each other in gene regulation 70-72. Particularly in cancer, for instance, in colorectal cancer (CRC), the removal of the H3K4me3 mark downregulates *METTL14*, reducing m^6^A accumulation on SOX4 mRNA, protecting it from degradation by YTHDF2, and leading to enhanced migration of CRC cells and SOX4-mediated epithelial-mesenchymal transition 73. Similarly, the m^6^A demethylation of lncRNA PVT1 by ALKBH5 promotes the development of osteosarcoma, highlighting the importance of chromatin modifiers in regulating m^6^A gene expression 74. Moreover, in myocardial injury, METTL3 can increase the m^6^A modification on HDAC4 (Histone deacetylase 4) mRNA, and IGF2BP1 can recognize these modification sites and enhance the stability of HDAC4 mRNA, thus exacerbating myocardial injury 75. Based on these studies, we postulate that crosstalk among epigenetic modifications might also exist in atherosclerosis, which could serve as an important direction for future research on epigenetic modifications in atherosclerosis.

Currently, certain progress has been made in therapeutic research targeting m^6^A modifications in atherosclerosis. Pemetrexed, the first-reported METTL4 antagonist, is capable of reducing the progression of atherosclerosis 76. However, due to its lack of cell-specificity *in vivo*, it failed to act selectively on the cells involved in the disease process, and this may potentially impact the physiological functions and metabolic processes of normal cells. Therefore, the results of this study contribute to the precise intervention of key regulators at the single-cell level. For example, drugs can be developed based on specific regulators in macrophages to modulate inflammation. These findings offer new insights into the role of m^6^A modification in atherosclerosis and novel targets for the prevention and treatment of this disease. Future studies should focus on developing specific inhibitors or modulators of these proteins and evaluating their efficacy in preclinical models of atherosclerosis.

## Conclusion

In this study, we identified the cell-type-specific regulatory mechanisms of m^6^A regulators in key atherosclerotic cell types. *ALKBH5* upregulated in ECs induced cell proliferation and migration involved in sprouting angiogenesis. *WTAP* specifically upregulated in SMCs promoted proliferation, migration and phenotypic transformation. The increased expressions of *METTL3* and* YTHDF2* in macrophages of atherosclerosis promoted the occurrence of inflammation. We further demonstrated that specific TFs influence cell types function by regulating the expression levels of m^6^A regulators. In addition, we discovered that m^6^A regulators mediated intercellular interactions through important ligand-receptor pairs. Overall, our research provides potential targets for future prevention and treatment of atherosclerosis.

## Materials and methods

### Cell culture and transfection

HCAECs (Cat. No. PAHX-C121; HyCyte) and HCASMCs (Cat. No. PAHX-C171; HyCyte) were cultured in Endothelial Cell Medium (ECM; Cat. No. 1001; ScienCell) and Smooth Muscle Cell Medium (SMCM; Cat. No. 1101; ScienCell) respectively, and cells at fifth to eighth passages were used for experiments. THP-1(Cat. No. TCH-C361; HyCyte) were cultured in Roswell Park Memorial Institute (RPMI) 1640 Medium (Cat. No. 8117072; Invitrogen, Thermo Fisher Scientific). HCAECs were treated in 6-well plates with either IL-1β (10 ng/mL; Cat. No. HZ-1164; Proteintech) or ox-LDL (50 µg/mL; Cat. No. 101300; Solarbio) for 48 hours. HCASMCs were treated in 6-well plates with ox-LDL (100 µg/mL; Cat. No. 101300; Solarbio) for 48 hours. THP-1 cells were seeded in 6-well plates (1×10^6^ cells/mL) and cultured with 100 ng/mL PMA (Cat. No. HY-18739, MCE) for 48 hours and then treated with ox-LDL (150 µg/mL; Cat. No. 101300; Solarbio) for 12 hours. The siRNA for siALKBH5 (Cat. No. stB0011723A-1-5, Ribobio), siWTAP (Cat. No. siB13822144850-1-5, Ribobio), siMETTL3(Cat. No. stB0012383A-1-5, Ribobio) and siControl (Cat. No. siN0000001-1-5, Ribobio) were performed using Lipofectamine RNAiMAX (Cat. No. 13778075, Invitrogen, Thermo Fisher Scientific) for 24 hours. Then, the supernatant was replaced by fresh medium and the cells were treated with different inflammatory factors.

### Real-time quantitative polymerase chain reaction (RT-qPCR)

Total RNA was isolated from cells using TRIzol reagent (Thermo Fisher Scientific, Invitrogen). 1 µg of total RNA was reverse-transcribed to cDNA with GoScript™ Reverse Transcription System (Promega). Resultant cDNA samples were subjected to qPCR on LightCycler® 480 Instrument II - Sequencing (Roche) using SYBR® qPCR Master Mix (Promega) and the primers as followed. β-actin levels were measured to serve as an internal control, and the relative expression levels were calculated using the 2-^ΔΔCt^ method.

The corresponding sequences of gene primers are as follows:

IL-1β Forward: 5'-TGGCTTATTACAGTGGCAATGAG-3',

Reverse: 5'-GTAGTGGTGGTCGGAGATTCG-3';

IL-6 Forward: 5'-AGCCACTCACCTCTTCAGAAC-3',

Reverse: 5'-GCAAGTCTCCTCATTGAATCCAG-3';

TNF-α Forward: 5'-GTCTGGGCAGGTCTACTTTGG-3',

Reverse: 5'-GAGGTTGAGGGTGTCTGAAG-3';

iNOS Forward: 5'-TCGGAACCAAGGATCACTGTG-3',

Reverse: 5'-AGGTGGAGGAGGGATGTGAC-3';

β-actin Forward: 5'-TCATGAAGTGTGTGACGTGGACATC-3',

Reverse: 5'-CAGCAGGAGCAATGATCTTGATCT-3'.

### Western blot

Protein was extracted from cells lysed in RIPA buffer (Cat. No. R0010, Solarbio) containing Protease Inhibitor Cocktail (Cat. No. HY-K0010, MCE). Protein concentrations were determined using the Pierce BCA Protein Assay Kit (Cat. No. 23227; Invitrogen, Thermo Fisher Scientific). Protein lysates were resolved on a 10% Bis-Tris gel (NP0303BOX; Invitrogen, Thermo Fisher Scientific) with NuPAGE MES SDS electrophoretic buffer and transferred to PVDF membranes. Membranes were blocked with 5% skim milk for 1 hour at room temperature and incubated with primary antibodies against ALKBH5 (Cat. No. A22137; ABclonal), KLF2 (Cat. No. 23384-1- AP; Proteintech), PLK2 (Cat. No. 15956-1-AP; Proteintech), CTCF (Cat. No. A19588; ABclonal), MYC (Cat. No. A19032; ABclonal), JUN (Cat. No. A25329; ABclonal), WTAP (Cat. No. A22751; ABclonal), PRKG1(Cat. No. A2565; ABclonal), MEF2C (Cat. No. A12385; ABclonal), FOS (Cat. No. A24619; ABclonal), METTL3 (Cat. No. 15073-1-AP; Proteintech), CX3CR1 (Cat. No. A2890; ABclonal), IRF1 (Cat. No. A7692; ABclonal) and GAPDH (Cat. No. 10494-1-AP; Proteintech) overnight at 4°C. Membranes were washed and incubated with HRP-conjugated secondary antibodies (Cat. No. SA00001-2; Proteintech) for 1 hour at room temperature. Protein signals were detected using an ECL western blot detection kit on a FUSION FX SPECTRA imaging system (Vilber, France). Band intensities were quantified using ImageJ software.

### Dot blot assays

Total RNA was extracted from HCAECs using TRIzol reagent (Cat. No. 15596018CN, Invitrogen, Thermo Fisher Scientific). RNA samples were doubly diluted to concentrations of 400 ng/μL, 200 ng/μL, and 100 ng/μL. RNAs were heat-treated at 95°C for 5 minutes and rapidly cooled on ice, and applied onto a Nylon transfer membrane (Cat. No. YA1760, Solarbio) using a vacuum suction system. Membranes were UV crosslinked, blocked, and incubated with an m^6^A-specific antibody (Cat. No. A19841, ABclonal) overnight at 4°C. After incubation with a secondary antibody conjugated to HRP for 1 hour at room temperature, signals were developed using an ECL western blot detection kit on a FUSION FX SPECTRA imaging system. A separate membrane was stained with methylene blue (MB) to serve as a loading control.

### Cell proliferation experiment

Cell proliferation was assessed using the YF®594 Click-iT EdU Imaging Kit (Cat. No. 40276ES60, Yeasen Biotechnology) following the manufacturer's instructions. Cells were incubated with an EdU mixture (10 μm) for 6 hours, fixed with 4% paraformaldehyde, decolorized with 2 mg/mL glycine, permeabilized with 0.5% TritonX-100, and washed with PBS. Cells were then incubated with the Click-iT reaction mixture (1×) for 30 minutes and stained with DAPI for 5 minutes. EdU-positive ECs were visualized and counted using confocal microscopy to capture images (LSM 980, Carl Zeiss) to evaluate cell proliferation.

### Scratch experiment

Cells were cultured in a 6-well plate to form a confluent monolayer. Straight-line scratch was created using a pipette tip. Cells were gently washed to remove debris and unattached cells. To promote migration over proliferation, we replaced with serum-free medium plates, and the healing of the scratch was observed at 0 and 6 hours using an inverted microscope (TI-BDTV2, Olympus). The scratch width was measured using ImageJ software to quantify cell migration.

### Migration experiment

A 24-well Boyden chamber with porous polycarbonate membrane (8 μm pore size; 3422; Corning) was used. Cells previously treated and grown to 70%-80% confluence in serum-free ECM medium were loaded onto the upper chamber. The lower chamber was filled with complete medium containing 10% FBS to induce migration for 24 hours. The upper surface was wiped with a cotton swab, and the cells on the lower surface were fixed with 4% formaldehyde and stained with crystal violet. The number of migratory cells was determined using ImageJ software.

### Tube formation assay

Matrigel (Cat. No. 40188ES08; Yeasen Biotechnology) was prepared on ice and refrigerated at 4°C. It was diluted 1:1 with ECM medium without FBS. 80 µL of diluted matrigel was added to each well of a 96-well plate and allowed to solidify for 1 hour at 37°C. Treated cells were incubated with calcein (Cat. No. 40719ES60; Yeasen Biotechnology, Shanghai, China) for 30 minutes, washed, and re-suspended. A 100 µL cell suspension (2 × 10^4^ cells/well) was applied to the matrigel-coated wells. After incubation at 37°C for 4 hours, non-adherent cells were removed by washing. Images were captured using a confocal microscopy (LSM 980, Carl Zeiss), and tube lengths and branches were measured using Image-Pro Plus software.

### Immunofluorescence staining experiment

HCAECs were fixed with 4% paraformaldehyde, blocked with 5% BSA at room temperature for 60 minutes and incubated with primary antibody of ALKBH5 at 4°C for 12 hours. The cells were incubated with a Goat anti-Rabbit IgG (H+L) Cross-Adsorbed Secondary Antibody, Alexa Fluor™ 647 (Cat. No. A-21244; Invitrogen, Thermo Fisher Scientific) at 37°C in the dark for 30 minutes, counterstained with DAPI. The cells were examined under a confocal microscopy (LSM 980, Carl Zeiss).

### Methylated RNA immunoprecipitation quantitative polymerase chain reaction (MeRIP-qPCR)

Frozen samples were homogenized with 0.5% NP-40 non-denatured lysate, but the RNA was not fragmented because of a primer design problem which we first used the SRAMP m^6^A modification site prediction tool to predict the target genes and designed primers for the abundant m^6^A modification sites. Briefly, the RNA was hybridized and mixed with Dynabead magnetic beads (Cat. 10001D, Thermo Fisher Scientific) and pre-labeled with IgG control antibodies (Cat. No. ab109489, Abcam) and Anti-m^6^A antibodies (Cat. No. ab208577, Abcam). DNase I (Cat. EN0521, Thermo Fisher Scientific) was used to digest the magnetic beads at room temperature, and Proteinase K (Cat. No. HY-K0010, MCE) was used to digest and eluate the RNA samples on magnetic beads. Then 1 ng of the total RNA and m^6^A IP RNA were used as templates in RT-qPCR, as described above.

The corresponding sequences of gene primers are as follows:

PLK2 Forward: 5'-GGACCCTATGGGACTCCTCT-3',

Reverse: 5'-CAGGCCACAGGGGAATTGTT-3';

KLF2 Forward: 5'-CCCCCTCCCAAACTGTGACT-3',

Reverse: 5'-TCGTGGTCTTTTCCCACCG-3';

PRKG1 Forward: 5'-GGGGTCACCATGATGCCTTT-3',

Reverse: 5'-GGTGAAAGGCTTTGCTTCAGG-3';

CX3CR1 Forward: 5'-TGATGGACCCAATGCACACA-3',

Reverse: 5'-AGGGCTCAGACACCCTTTTG-3'.

### Patient samples

Our scRNA-seq data of 8 samples from coronary artery sites of 4 patients who underwent heart transplantation were obtained from GEO database (https://www.ncbi.nlm.nih.gov/geo/) in GSE131778 77. Clinical information collected included sample names and tissue.

### scRNA-seq data processing and cell types identification

FASTQ files were processed using Cell Ranger software (version 6.1.2) from 10x Genomics to perform cellular barcode de-multiplexing and read alignment to the prebuilt GRCh38 reference genome. We generated an expression matrix of 18,411 cells after integrating the raw data. Next, we intersected the raw data expression matrix with the expression matrix provided in the article. The R package Seurat (version 4.3.1) was used to process the UMI count matrix 78. After preliminary data processing, we obtained an expression matrix containing 11,756 cells. Genes covered by fewer than six cells were filtered out from the data. We excluded cells with the number of detected genes < 200 or > 3,500, as well as cells with mitochondrial gene expression proportion > 7%. The remaining 11,722 cells were used for further analysis. The top 2,000 genes with the highest cell-to-cell variance were identified through the function “FindVariableFeatures”. A shared nearest neighbour graph was constructed using 50 dimensions of the principal components (PCs). Clustering was then performed using “FindClusters” function with a resolution of 0.5, which were visualized by t-SNE. Marker genes for each cluster were determined using “FindAllMarkers” function with log_2_FC (log_2_Fold change) ≥ 0.25.

### Construction of metacell maps

The R package hdWGCNA (version 0.2.19) was used to perform the MetaCell method on the main cell types. The idents of the metacell seurat object were defined as “cell type” and “donor” to determine the group in which metacells will be constructed. Different k-values were assigned based on the number of cells for SMCs (k = 20), ECs (k = 20) and macrophages (k = 15). The number of neighbors for each cell is limited by k-value. Based on the gene expression matrix of metacells, we calculated the correlation between m^6^A regulators with pathways and genes by Pearson correlation, as well as differential analysis between two groups with high and low expression of m^6^A regulators.

### Identification of DEGs

Differential gene expression analysis was performed with the “FindMarkers” function of Seurat between m^6^A regulators (WTAP, ALKBH5, METTL3, YTHDF2)-high and low groups using Wilcox test. Only those with *P* < 0.05 and |log_2_FC| ≥ 0.2 were identified as DEGs. GO enrichment analysis was performed by clusterProfiler (version 4.8.3) and enrichR (version 3.2) to conduct biological process enrichment analysis with the DEGs of each group. The results were visualized using ggplot2 (version 3.4.4).

### Gene set enrichment analysis

GSEA was performed to examine the significant differences in predefined gene sets within different cell types (SMCs, ECs, macrophages) between high and low expression groups of m^6^A regulators (WTAP, ALKBH5, METTL3, YTHDF2), which was performed based on clusterProfiler. GO gene sets were obtained from the Molecular Signatures Database (version 7.5.1).

### Gene set variation analysis (GSVA)

GSVA was utilized to estimate the signature scores of predefined biological pathways or gene sets across different conditions. We employed the GSVA function from the GSVA package (version 1.48.3) to assign signature scores to m^6^A regulators and disease-associated pathways within the principal cell types in atherosclerosis 79, followed by Spearman correlation analysis to investigate the relationships between m^6^A regulators and pathways in different cell types (Figure S1D).

### SCENIC analysis

We used SCENIC (version 1.2.4) to analyze TFs with potential regulatory roles and their target genes in different cell types within the plaques 80. For the python implementation of the SCENIC algorithm (pySCENIC) (version 0.11.2), we used scRNA data from different cell types as input. GENIE3 (version 1.22.0) inferred TFs and their target genes based on analyzing correlations in gene expression across cells. RcisTarget (version 1.20.0) was utilized to identify enriched transcription factor-binding motifs and to predict candidate target genes (regulons). Finally, the activity of a regulon was measured using AUCell (version 1.22.0). A high AUCell value indicates a higher level of activity and enrichment of the regulon. The regulon-group heatmap was generated with pheatmap (version 1.0.12). The TF module network was visualized through Cytoscape (version 3.10.1) or ggplot2.

### Identification of co-expressed gene modules for m^6^A regulators

To identify the co-expressed gene modules of m^6^A regulators, we performed hdWGCNA (version 0.2.19) analysis using default parameters unless otherwise noted 34. We created metacells by aggregating single-cell transcriptomes and pooling cells within the same cell type to address the sparsity of single-cell data, which retained the necessary metadata for hdWGCNA. Furthermore, we generated an expression matrix for metacells and applied a soft threshold. Soft thresholding was used to reduce gene-gene correlation noise. By calculating the pairwise correlation of the input genes, a topological overlap matrix was obtained after the transformation. Eigengene-based connectivity (kME) parameters from the hdWGCNA package were used to define hub genes. The modules obtained through hdWGCNA were scored within cell types using AUCell and then subjected to correlation analysis with the pathways of interest. Additionally, we performed functional enrichment of important gene modules based on “RunEnrichr” function.

### SMCs subclustering analysis

After annotation of the major cell clusters, we selected SMCs for further clustering. To identify cell subclusters, we subset the gene matrix of SMCs from the entire matrix and performed the analysis. 2,000 new variable genes were identified and used for principal component analysis. These genes captured the cell-to-cell variation within the defined cell population and were distinct from those in the whole dataset. Scaling was performed based on all genes in SMCs. The first 15 PCs were determined as significant PCs and were used for downstream analysis. Subsequently, Clustering was then performed using Seurat function “FindClusters” with a resolution of 0.3.

### Constructing single cell trajectories in SMCs

Single-cell trajectory analysis was performed using Monocle2 (version 2.28.0) package to identify developmental transitions in SMCs 81. We used DEGs identified by Seurat to sort cells in pseudotime order. The actual evolutionary time of each cell told us the start point of pseudotime in the second round of “orderCells”. We then set this state to the “root_state” parameter and called “orderCells” again. Dimension reduction was conducted by using “DDRTree” and visualized using “plot_cell_trajectory” function. We used AUCell to assess signatures of disease-related functional pathways to evaluate changes in gene sets during the evolution of SMC subclusters.

### Cell-cell communication analysis

Communication between ECs or macrophages and other cell types was quantified using CellChat (version 1.6.1) 82. Differences between groups were compared by dividing cells according to the high or low expression of m^6^A regulators (ALKBH5, METTL3, YTHDF2). Specifically, we examined the overall count of interactions and the changes in communication between particular cell types across different groups. We then identified differences in signaling pathways by comparing overall information flow between two groups. Additionally, the “netVisual_bubble” function was used to present bubble plots of significant ligand-receptor interactions between ligand cell types and receptor cell types, with the “remove.isolate” parameter included set to “TRUE”.

### Quantification and statistical analysis

All data were analyzed using GraphPad Software's PRISM (version 9), and the results were presented as the mean±SD. Comparisons between groups were conducted using two-tailed Wilcoxon rank-sum test or Student's t-test. *P* < 0.05 was considered statistically significant. The corresponding *P* values are presented in the indicated figures, and the levels of significance are denoted as follows: *P < 0.05; **P < 0.01; ***P < 0.001.

## Supplementary Material

Supplementary figures.

## References

[1] Björkegren JLM, Lusis AJ (2022). Atherosclerosis: recent developments. Cell.

[2] Libby P, Buring JE, Badimon L, Hansson GK, Deanfield J, Bittencourt MS (2019). Atherosclerosis. Nat Rev Dis Primers.

[3] Stroope C, Nettersheim FS, Coon B, Finney AC, Schwartz MA, Ley K (2024). Dysregulated cellular metabolism in atherosclerosis: mediators and therapeutic opportunities. Nat Metab.

[4] Lin A, Miano JM, Fisher EA, Misra A (2024). Chronic inflammation and vascular cell plasticity in atherosclerosis. Nat Cardiovasc Res.

[5] Hahn C, Schwartz MA (2009). Mechanotransduction in vascular physiology and atherogenesis. Nat Rev Mol Cell Biol.

[6] Worssam MD, Jørgensen HF (2021). Mechanisms of vascular smooth muscle cell investment and phenotypic diversification in vascular diseases. Biochem Soc Trans.

[7] Arvanitis M, Lowenstein CJ (2023). Dyslipidemia. Ann Intern Med.

[8] Reith C, Armitage J (2016). Management of residual risk after statin therapy. Atherosclerosis.

[9] Williams JW, Winkels H, Durant CP, Zaitsev K, Ghosheh Y, Ley K (2020). Single cell RNA sequencing in atherosclerosis research. Circ Res.

[10] de Winther MPJ, Bäck M, Evans P, Gomez D, Goncalves I, Jørgensen HF (2023). Translational opportunities of single-cell biology in atherosclerosis. Eur Heart J.

[11] an H, Xue C, Auerbach BJ, Fan J, Bashore AC, Cui J (2020). Single-cell genomics reveals a novel cell state during smooth muscle cell phenotypic switching and potential therapeutic targets for atherosclerosis in mouse and human. Circulation.

[12] Willemsen L, de Winther MP (2020). Macrophage subsets in atherosclerosis as defined by single-cell technologies. J Pathol.

[13] Zernecke A, Winkels H, Cochain C, Williams JW, Wolf D, Soehnlein O (2020). Meta-analysis of leukocyte diversity in atherosclerotic mouse aortas. Circ Res.

[14] Zernecke A, Erhard F, Weinberger T, Schulz C, Ley K, Saliba AE (2023). Integrated single-cell analysis-based classification of vascular mononuclear phagocytes in mouse and human atherosclerosis. Cardiovasc Res.

[15] Lin JD, Nishi H, Poles J, Niu X, Mccauley C, Rahman K (2019). Single-cell analysis of fate-mapped macrophages reveals heterogeneity, including stem-like properties, during atherosclerosis progression and regression. JCI Insight.

[16] Fernandez DM, Rahman AH, Fernandez NF, Chudnovskiy A, Amir ED, Amadori L (2019). Single-cell immune landscape of human atherosclerotic plaques. Nat Med.

[17] Hu Z, Liu W, Hua X, Chen X, Chang Y, Hu Y (2021). Single-cell transcriptomic atlas of different human cardiac arteries identifies cell types associated with vascular physiology. Arterioscler Thromb Vasc Biol.

[18] Pan H, Xue C, Auerbach BJ, Fan J, Bashore AC, Cui J (2020). Single-cell genomics reveals a novel cell state during smooth muscle cell phenotypic switching and potential therapeutic targets for atherosclerosis in mouse and human. Circulation.

[19] Zhao G, Lu H, Liu Y, Zhao Y, Zhu T, Garcia-Barrio MT (2021). Single-cell transcriptomics reveals endothelial plasticity during diabetic atherogenesis. Front Cell Dev Biol.

[20] Khyzha N, Alizada A, Wilson MD, Fish JE (2017). Epigenetics of atherosclerosis: emerging mechanisms and methods. Trends Mol Med.

[21] Tan Q, He S, Leng X, Zheng D, Mao F, Hao J (2022). The mechanism and role of N^6^-methyladenosine (m^6^A) modification in atherosclerosis and atherosclerotic diseases. J Cardiovasc Dev Dis.

[22] Xu Z, Lv B, Qin Y, Zhang B (2022). Emerging roles and mechanism of m^6^A methylation in cardiometabolic diseases. Cells.

[23] Xu S, Pelisek J, Jin ZG (2018). Atherosclerosis is an epigenetic disease. Trends Endocrinol Metab.

[24] Liu S, Cao Y, Zhang Y (2024). Regulatory roles of RNA methylation in vascular lesions in ocular and cardiopulmonary diseases. Crit Rev Clin Lab Sci.

[25] Nossent AY (2023). The epitranscriptome: RNA modifications in vascular remodelling. Atherosclerosis.

[26] Wang L, Huang Z, Huang W, Chen X, Shan P, Zhong P (2017). Inhibition of epidermal growth factor receptor attenuates atherosclerosis via decreasing inflammation and oxidative stress. Sci Rep.

[27] Li B, Zhang T, Liu M, Cui Z, Zhang Y, Liu M (2022). RNA N^6^-methyladenosine modulates endothelial atherogenic responses to disturbed flow in mice. Elife.

[28] Jian D, Wang Y, Jian L, Tang H, Rao L, Chen K (2020). METTL14 aggravates endothelial inflammation and atherosclerosis by increasing FOXO1 N^6^-methyladeosine modifications. Theranostics.

[29] Mo C, Yang M, Han X, Li J, Gao G, Tai H (2017). Fat mass and obesity-associated protein attenuates lipid accumulation in macrophage foam cells and alleviates atherosclerosis in apolipoprotein E-deficient mice. J Hypertens.

[30] Stelzer G, Rosen N, Plaschkes I, Zimmerman S, Twik M, Fishilevich S (2016). The GeneCards suite: from gene data mining to disease genome sequence analyses. Curr Protoc Bioinformatics.

[31] Lamalice L, Le Boeuf F, Huot J (2007). Endothelial cell migration during angiogenesis. Circ Res.

[32] Litwin M, Radwańska A, Paprocka M, Kieda C, Dobosz T, Witkiewicz W (2015). The role of FGF2 in migration and tubulogenesis of endothelial progenitor cells in relation to pro-angiogenic growth factor production. Mol Cell Biochem.

[33] Langfelder P, Horvath S (2008). WGCNA: an R package for weighted correlation network analysis. BMC Bioinformatics.

[34] Morabito S, Reese F, Rahimzadeh N, Miyoshi E, Swarup V (2023). hdWGCNA identifies co-expression networks in high-dimensional transcriptomics data. Cell Rep Methods.

[35] Shi Y, Xu X, Zhang Q, Fu G, Mo Z, Wang GS (2014). tRNA synthetase counteracts c-Myc to develop functional vasculature. Elife.

[36] Gimbrone MA Jr, García-Cardeña G (2016). Endothelial cell dysfunction and the pathobiology of atherosclerosis. Circ Res.

[37] Hajra L, Evans AI, Chen M, Hyduk SJ, Collins T, Cybulsky MI (2000). The NF-kappa B signal transduction pathway in aortic endothelial cells is primed for activation in regions predisposed to atherosclerotic lesion formation. Proc Natl Acad Sci U S A.

[38] Howe KL, Cybulsky M, Fish JE (2022). The endothelium as a hub for cellular communication in atherogenesis: is there directionality to the message?. Front Cardiovasc Med.

[39] Lu YW, Lowery AM, Sun LY, Singer HA, Dai G, Adam AP (2017). Endothelial myocyte enhancer factor 2c inhibits migration of smooth muscle cells through fenestrations in the internal elastic lamina. Arterioscler Thromb Vasc Biol.

[40] Miao G, Zhao X, Chan SL, Zhang L, Li Y, Zhang Y (2022). Vascular smooth muscle cell c-Fos is critical for foam cell formation and atherosclerosis. Metabolism.

[41] Lv D, Meng D, Zou FF, Fan L, Zhang P, Yu Y (2011). Activating transcription factor 3 regulates survivability and migration of vascular smooth muscle cells. IUBMB Life.

[42] Bennett MR, Sinha S, Owens GK (2016). Vascular smooth muscle cells in atherosclerosis. Circ Res.

[43] Frismantiene A, Philippova M, Erne P, Resink TJ (2018). Smooth muscle cell-driven vascular diseases and molecular mechanisms of VSMC plasticity. Cell Signal.

[44] Mao C, Ma Z, Jia Y, Li W, Xie N, Zhao G (2021). Nidogen-2 maintains the contractile phenotype of vascular smooth muscle cells and prevents neointima formation via bridging jagged1-notch3 signaling. Circulation.

[45] Yoshida T, Kaestner KH, Owens GK (2008). Conditional deletion of Krüppel-like factor 4 delays downregulation of smooth muscle cell differentiation markers but accelerates neointimal formation following vascular injury. Circ Res.

[46] Qiao L, Zhang X, Liu M, Liu X, Dong M, Cheng J (2017). Ginsenoside Rb1 enhances atherosclerotic plaque stability by improving autophagy and lipid metabolism in macrophage foam cells. Front Pharmacol.

[47] Johnston JM, Angyal A, Bauer RC, Hamby S, Suvarna SK, Baidžajevas K (2019). Myeloid tribbles 1 induces early atherosclerosis via enhanced foam cell expansion. Sci Adv.

[48] Zheng X, Zhou B, Li Y, Zhong H, Huang Z, Gu M (2023). Transcriptome-wide N^6^-methyladenosine methylation profile of atherosclerosis in mice. BMC Genomics.

[49] Zaina S, Heyn H, Carmona FJ, Varol N, Sayols S, Condom E (2014). DNA methylation map of human atherosclerosis. Circ Cardiovasc Genet.

[50] Xu S, Ilyas I, Little PJ, Li H, Kamato D, Zheng X (2021). Endothelial dysfunction in atherosclerotic cardiovascular diseases and beyond: from mechanism to pharmacotherapies. Pharmacol Rev.

[51] Zhang BY, Han L, Tang YF, Zhang GX, Fan XL, Zhang JJ (2020). METTL14 regulates m^6^A methylation-modified primary miR-19a to promote cardiovascular endothelial cell proliferation and invasion. Eur Rev Med Pharmacol Sci.

[52] Depuydt MAC, Prange KHM, Slenders L, Örd T, Elbersen D, Boltjes A (2020). Microanatomy of the human atherosclerotic plaque by single-cell transcriptomics. Circ Res.

[53] Basatemur GL, Jørgensen HF, Clarke MCH, Bennett MR, Mallat Z (2019). Vascular smooth muscle cells in atherosclerosis. Nat Rev Cardiol.

[54] Chen J, Lai K, Yong X, Yin H, Chen Z, Wang H (2023). Silencing METTL3 stabilizes atherosclerotic plaques by regulating the phenotypic transformation of vascular smooth muscle cells via the miR-375-3p/PDK1 axis. Cardiovasc Drugs Ther.

[55] Tabas I, Bornfeldt KE (2016). Macrophage phenotype and function in different stages of atherosclerosis. Circ Res.

[56] Zhang X, Li X, Jia H, An G, Ni J (2021). The m^6^A methyltransferase METTL3 modifies PGC-1α mRNA promoting mitochondrial dysfunction and oxLDL-induced inflammation in monocytes. J Biol Chem.

[57] Chien CS, Li JY, Chien Y, Wang ML, Yarmishyn AA, Tsai PH (2021). METTL3-dependent N^6^-methyladenosine RNA modification mediates the atherogenic inflammatory cascades in vascular endothelium. Proc Natl Acad Sci U S A.

[58] Zheng L, Chen X, Yin Q, Gu J, Chen J, Chen M (2022). RNA-m^6^A modification of HDGF mediated by Mettl3 aggravates the progression of atherosclerosis by regulating macrophages polarization via energy metabolism reprogramming. Biochem Biophys Res Commun.

[59] Zheng Y, Li Y, Ran X, Wang D, Zheng X, Zhang M (2022). Mettl14 mediates the inflammatory response of macrophages in atherosclerosis through the NF-κB/IL-6 signaling pathway. Cell Mol Life Sci.

[60] Ma S, Sun B, Duan S, Han J, Barr T, Zhang J (2023). YTHDF2 orchestrates tumor-associated macrophage reprogramming and controls antitumor immunity through CD8^+^ T cells. Nat Immunol.

[61] Dong L, Chen C, Zhang Y, Guo P, Wang Z, Li J (2021). The loss of RNA N^6^-adenosine methyltransferase Mettl14 in tumor-associated macrophages promotes CD8^+^ T cell dysfunction and tumor growth. Cancer Cell.

[62] Yu L, Zhang J, Gao A, Zhang M, Wang Z, Yu F (2022). An intersegmental single-cell profile reveals aortic heterogeneity and identifies a novel Malat1^+^ vascular smooth muscle subtype involved in abdominal aortic aneurysm formation. Signal Transduct Target Ther.

[63] Viswanathan G, Kirshner HF, Nazo N, Ali S, Ganapathi A, Cumming I (2023). Single-cell analysis reveals distinct immune and smooth muscle cell populations that contribute to chronic thromboembolic pulmonary hypertension. Am J Respir Crit Care Med.

[64] Gomez D, Owens GK (2012). Smooth muscle cell phenotypic switching in atherosclerosis. Cardiovasc Res.

[65] Ben Hassine A, Petit C, Thomas M, Mundweiler S, Guignandon A, Avril S (2024). Gene expression modulation in human aortic smooth muscle cells under induced physiological mechanical stretch. Sci Rep.

[66] Cai Y, Yu R, Zhang Z, Li D, Yi B, Feng Z (2023). Mettl3/Ythdf2 regulate macrophage inflammation and ROS generation by controlling Pyk2 mRNA stability. Immunol Lett.

[67] Zampetaki A, Zeng L, Margariti A, Xiao Q, Li H, Zhang Z (2010). Histone deacetylase 3 is critical in endothelial survival and atherosclerosis development in response to disturbed flow. Circulation.

[68] Dai Y, Chen D, Xu T (2022). DNA methylation aberrant in atherosclerosis. Front Pharmacol.

[69] Connelly JJ, Cherepanova OA, Doss JF, Karaoli T, Lillard TS, Markunas CA (2013). Epigenetic regulation of *COL15A1* in smooth muscle cell replicative aging and atherosclerosis. Hum Mol Genet.

[70] Liu ZY, Lin LC, Liu ZY, Yang JJ, Tao H (2024). m^6^A epitranscriptomic and epigenetic crosstalk in cardiac fibrosis. Mol Ther.

[71] Bove G, Amin S, Babaei M, Benedetti R, Nebbioso A, Altucci L (2023). Interplay between m^6^A epitranscriptome and epigenome in cancer: current knowledge and therapeutic perspectives. Int J Cancer.

[72] Kan RL, Chen J, Sallam T (2022). Crosstalk between epitranscriptomic and epigenetic mechanisms in gene regulation. Trends Genet.

[73] Chen X, Xu M, Xu X, Zeng K, Liu X, Pan B (2020). METTL14-mediated N^6^-methyladenosine modification of SOX4 mRNA inhibits tumor metastasis in colorectal cancer. Mol Cancer.

[74] Chen S, Zhou L, Wang Y (2020). ALKBH5-mediated m^6^A demethylation of lncRNA PVT1 plays an oncogenic role in osteosarcoma. Cancer Cell Int.

[75] Shen H, Xie K, Li M, Yang Q, Wang X (2022). N^6^-methyladenosine (m^6^A) methyltransferase METTL3 regulates sepsis-induced myocardial injury through IGF2BP1/HDAC4 dependent manner. Cell Death Discov.

[76] Zheng L, Chen X, He X, Wei H, Li X, Tan Y (2024). METTL4-Mediated Mitochondrial DNA N^6^-methyldeoxyadenosine promoting macrophage inflammation and atherosclerosis. Circulation.

[77] Wirka RC, Wagh D, Paik DT, Pjanic M, Nguyen T, Miller CL (2019). Atheroprotective roles of smooth muscle cell phenotypic modulation and the *TCF21* disease gene as revealed by single-cell analysis. Nat Med.

[78] Butler A, Hoffman P, Smibert P, Papalexi E, Satija R (2018). Integrating single-cell transcriptomic data across different conditions, technologies, and species. Nat Biotechnol.

[79] Hänzelmann S, Castelo R, Guinney J (2013). GSVA: gene set variation analysis for microarray and RNA-seq data. BMC Bioinformatics.

[80] Aibar S, González-Blas CB, Moerman T, Huynh-Thu VA, Imrichova H, Hulselmans G (2017). SCENIC: single-cell regulatory network inference and clustering. Nat Methods.

[81] Qiu X, Mao Q, Tang Y, Wang L, Chawla R, Pliner HA (2017). Reversed graph embedding resolves complex single-cell trajectories. Nat Methods.

[82] Jin S, Guerrero-Juarez CF, Zhang L, Chang I, Ramos R, Kuan CH (2021). Inference and analysis of cell-cell communication using CellChat. Nat Commun.

