# Supplementary material for: Deciphering single-cell landscape unravels cell-type-specific functional roles of RNA m6A modification in atherosclerosis
Source: Theranostics. 2025 Mar 29;15(10):4785–807. doi: 10.7150/thno.104179 (PMC11984397; doi:10.7150/thno.104179)
Supplement: Supplementary file 1 — Supplementary figures. [file thnov15p4785s1.pdf]

Supplementary Materials for

Deciphering single-cell landscape unravels cell-type-specific functional roles of RNA m^{6}A modification in atherosclerosis

Xiaorui Ping[1]†, Xiaoyun Liang[2,3,4,5]†, Wenlu Xing[1]†, Saiqi Wang[1]†, Fengcongzhe Gong[1], Yaqi Cheng[1], Songqi Duan[1,6], Xueqi Lv[1], Xueying Li[1], Tianli Zhang[1], Chunxiao Chen[1], Yuxin Zhang[1], Chengzhu Yuan[1], Shangyu Liu[2,3,4,5*], Gang Liu[2,3,4,5*] and Baofa Sun[1*]

Corresponding author: sunbf@nankai.edu.cn; cardio2004@hebmu.edu.cn; liushangyu_fw@163.com;

The PDF file includes:

Figures S1 to S11

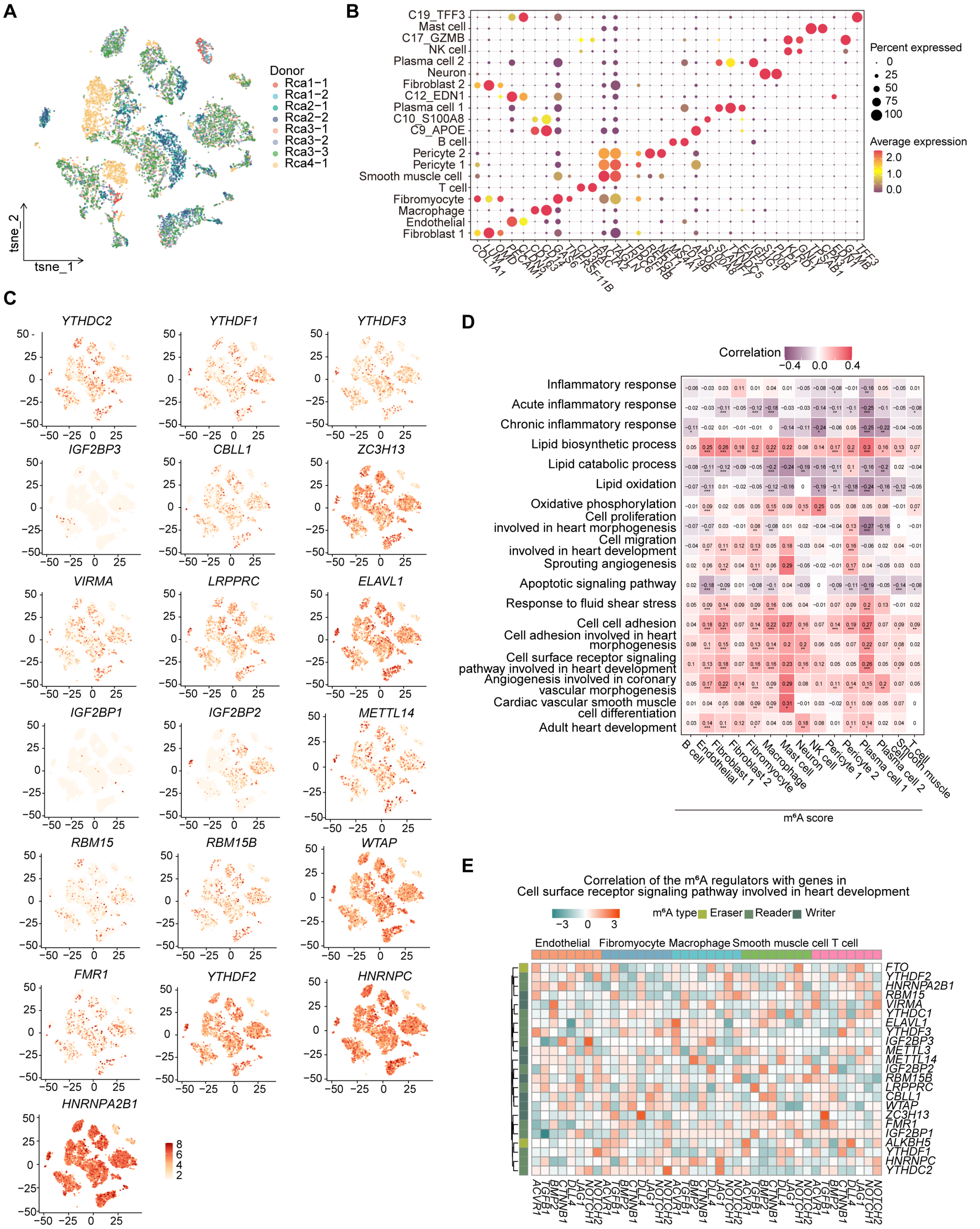

Figure S1. The distribution of m⁶A regulators in all cell types and correlations between m⁶A regulators and disease-related pathways in atherosclerosis. (A) The t-SNE projection of 11,722 single cells from atherosclerotic patients. Each point represents a single cell, colored by donor. Rca (Right coronary artery). (B) The scaled expression of marker genes across different cell types. (C) T-SNE projections of 20

clusters, with each cell colored based on the relative normalized expression of m^6A regulators. (D) Heatmap showing the correlations between m^6A regulators and signatures of diseased-related pathways. *P* value was calculated by Spearman correlation. (E) Correlation analysis between the expression levels of m^6A regulators and genes in "Cell surface receptor signaling pathway involved in heart development" in key cell types. The color depth of each cell represents the strength of correlation between different m^6A regulators and genes in different cell types.

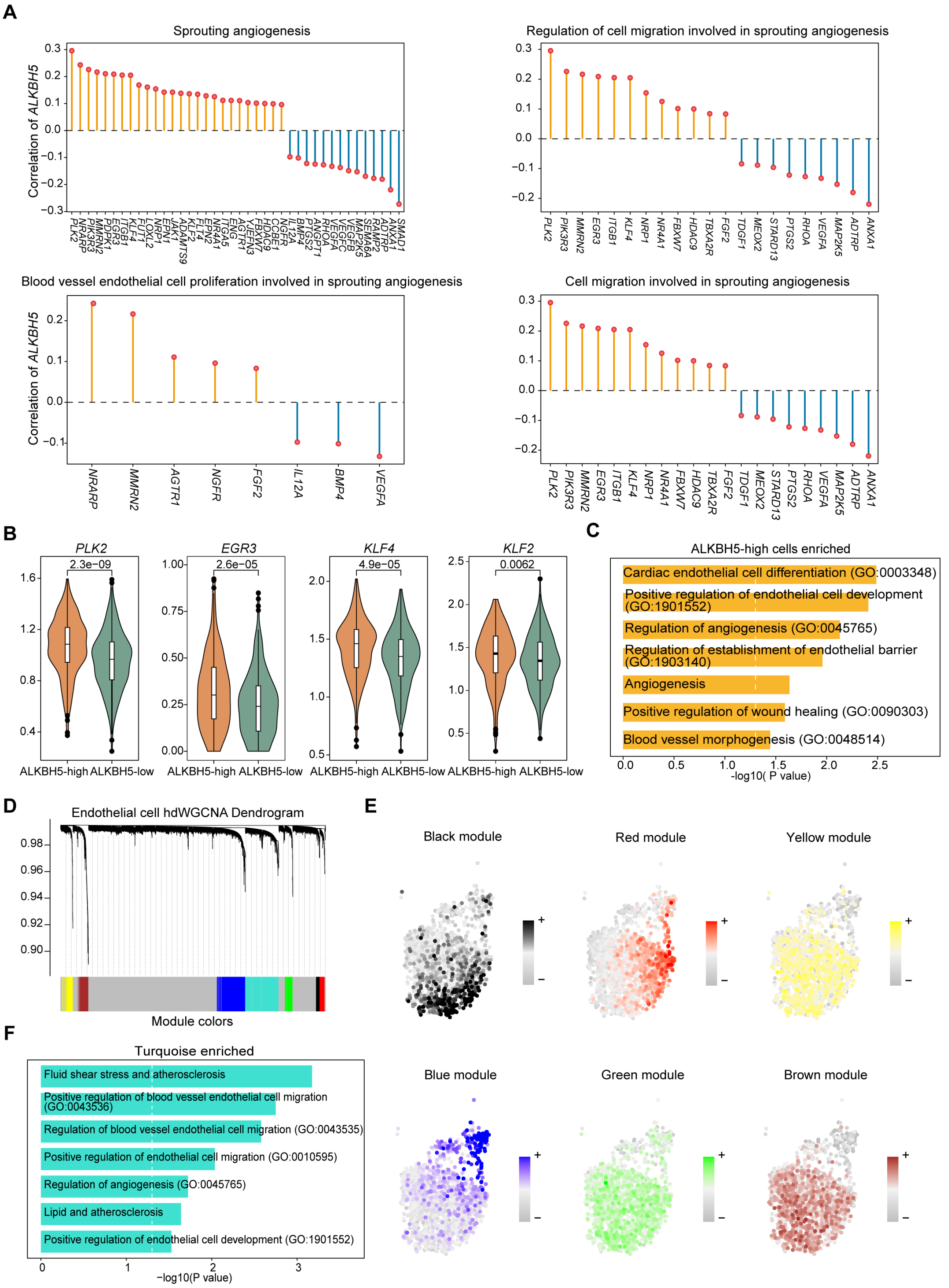

Figure S2. Correlations of *ALKBH5* and pathway genes, as well as the function of turquoise module. (A) The correlations between *ALKBH5* and genes enriched in sprouting angiogenesis pathways. The yellow lines represent positive correlation, while the blue lines represent negative correlation. (B) The expression of genes related to

sprouting angiogenesis in ALKBH5-high group and ALKBH5-low group. (C) Disease-related GO terms and pathways enriched by ALKBH5-high group cells. (D) ECs-related modules in atherosclerosis generated by hdWGCNA. (E) Module feature plots showing the distribution of different modules. Sorted based on the values of hMEs, with the color depth of the points indicating the level of hMEs values. (F) Disease-related pathways enriched by *ALKBH5* co-expressed genes within the turquoise module.

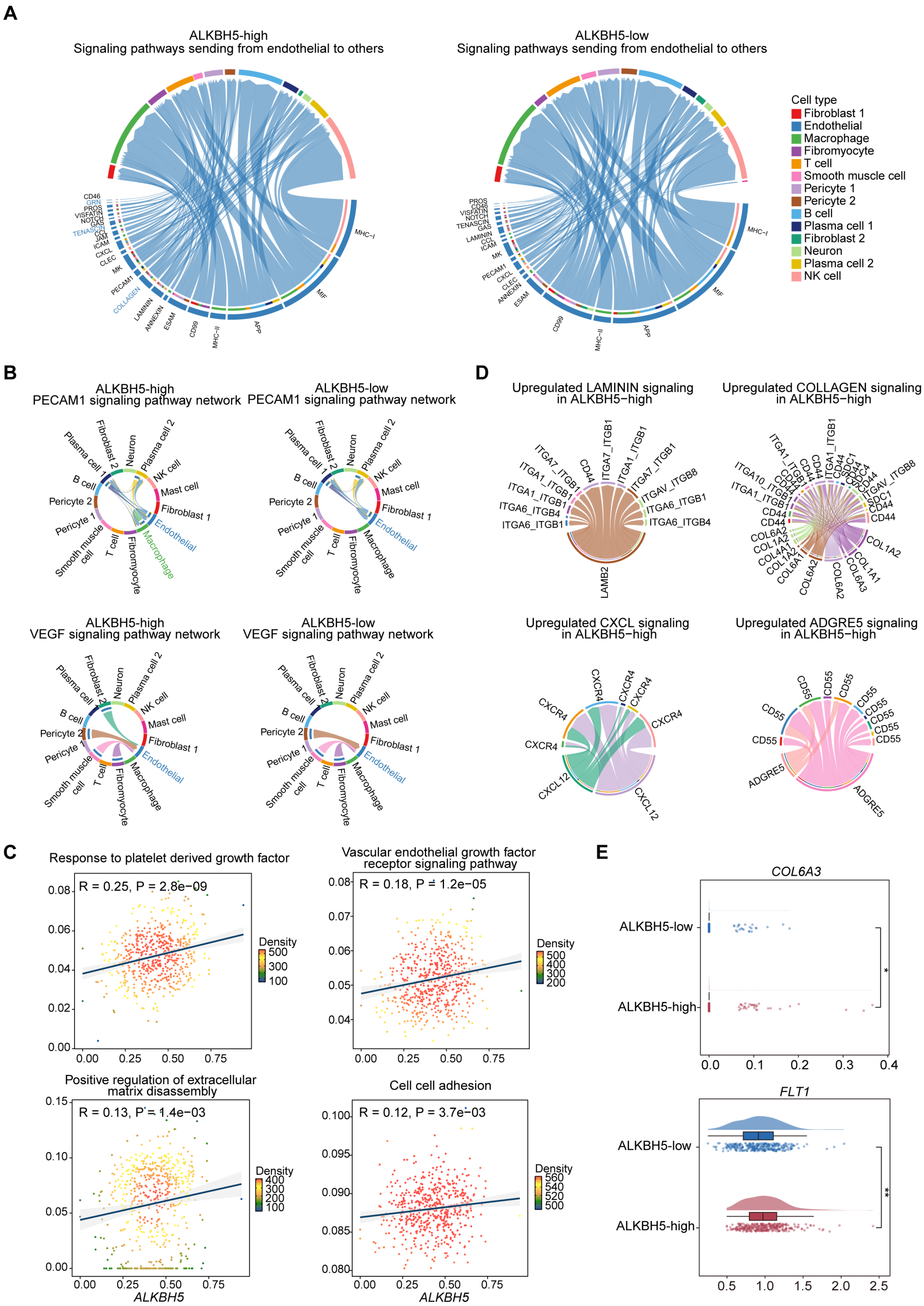

Figure S3. Comparison of ECs intercellular communication between ALKBH5-high and ALKBH5-low groups. (A) Chord plots showing the network of signal flows emanating from ECs as ligand cells in ALKBH5-high and ALKBH5-low groups. Key signaling pathways in ALKBH5-high group are colored by blue. (B) Chord plots

showing signaling pathways (PECAM1, VEGF) of cell-cell communication networks at the signaling pathway level in ALKBH5-high and ALKBH5-low groups. (C) Correlations between *ALKBH5* expression and signatures of pathways involved in cell adhesion and angiogenesis of ECs. (D) Chord diagrams showing the upregulated ligand-receptor pairs of signaling pathways (LAMININ, COLLAGEN, CXCL, ADGRE5). (E) Violin plots showing the expression levels of ligand and receptor genes in ALKBH5-high group and ALKBH5-low group. The statistical differences between the groups were determined through Wilcoxon rank test.

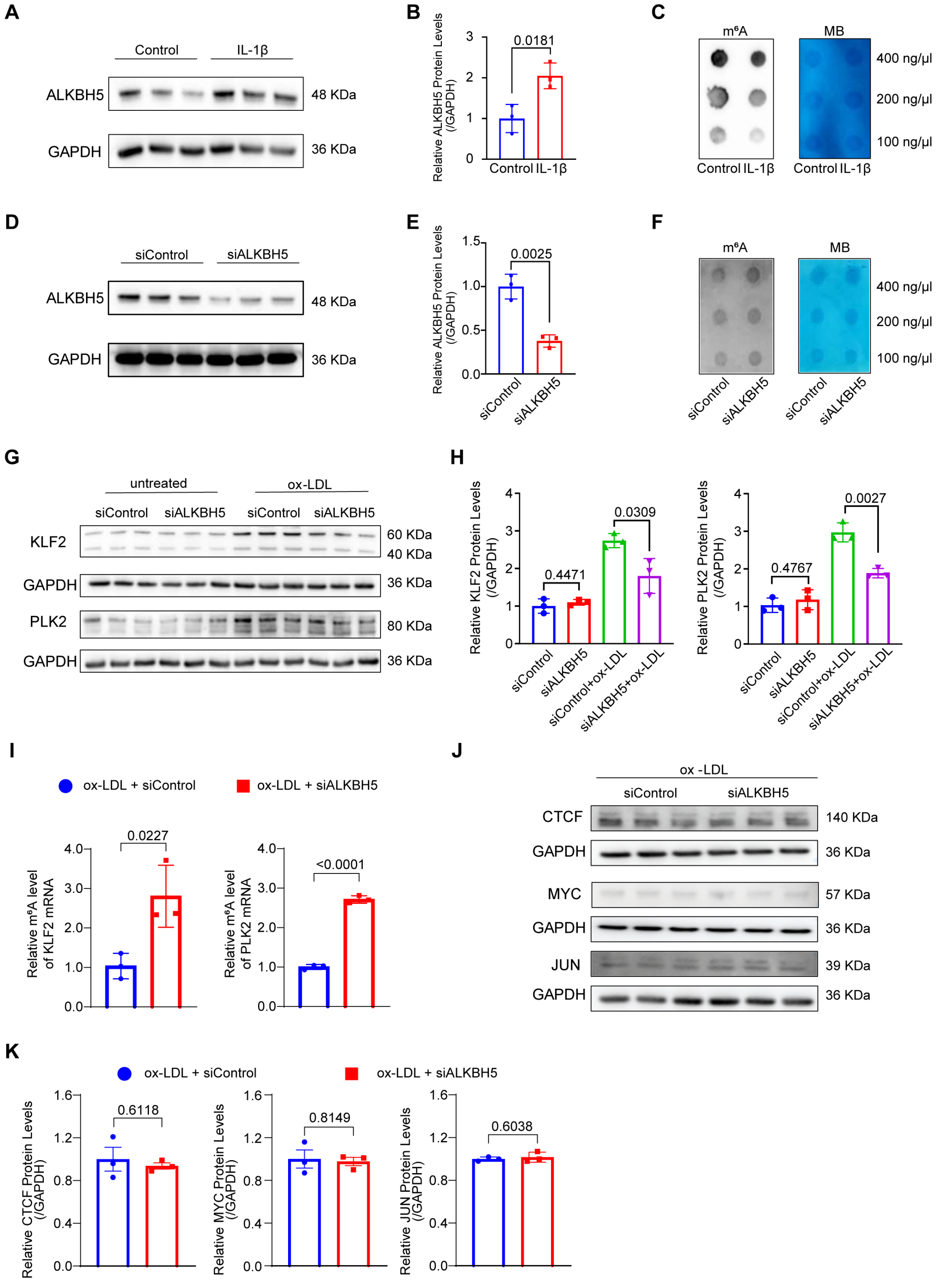

Figure S4. ALKBH5 upregulation in HCAECs under inflammatory factor stimulation is associated with enhanced angiogenesis. (A) Representative western blot images of ALKBH5 levels in control and HCAECs treated with 10 nmol IL-1β at 48 hours. (B) Quantification of ALKBH5 levels in control and HCAECs treated with

10 nmol IL-1β at 48 hours (n=3). (C) Dot blot assay using an anti-m^6A antibody in 10 ng/mL IL-1β-stimulated HCAECs. MB staining was included as a loading control. Total RNA concentration: 400 ng/μL, 200 ng/μL, 100 ng/μL. (D) Representative western blot images of ALKBH5 levels after the ALKBH5 siRNA (siALKBH5) and control siRNA (siControl) transfection without any delay. (E) Representative quantification of ALKBH5 levels after siALKBH5 and siControl transfection (n=3). (F) Dot blot assay using an anti-m^6A antibody in siALKBH5 and siControl HCAECs. MB staining was included as a loading control. (G) Representative western blot images of KLF2 and PLK2 levels in siControl and siALKBH5 HCAECs with or without ox-LDL stimulation. (H) Representative quantification of KLF2 and PLK2 levels in siControl and siALKBH5 HCAECs with or without ox-LDL stimulation (n=3). (I) MeRIP-qPCR analysis of KLF2 and PLK2 m^6A in ox-LDL-induced HCAECs treated with siControl or siALKBH5 (n=3). (J) Representative western blot images of CTCF, MYC, JUN levels in HCAECs transfected with siControl or siALKBH5 with ox-LDL at 48 hours. (K) Quantification of CTCF, MYC, JUN in HCAECs transfected with siControl or siALKBH5 with ox-LDL at 48 hours (n=3). Data are presented as mean±SD. T test followed by a Normality test was used.

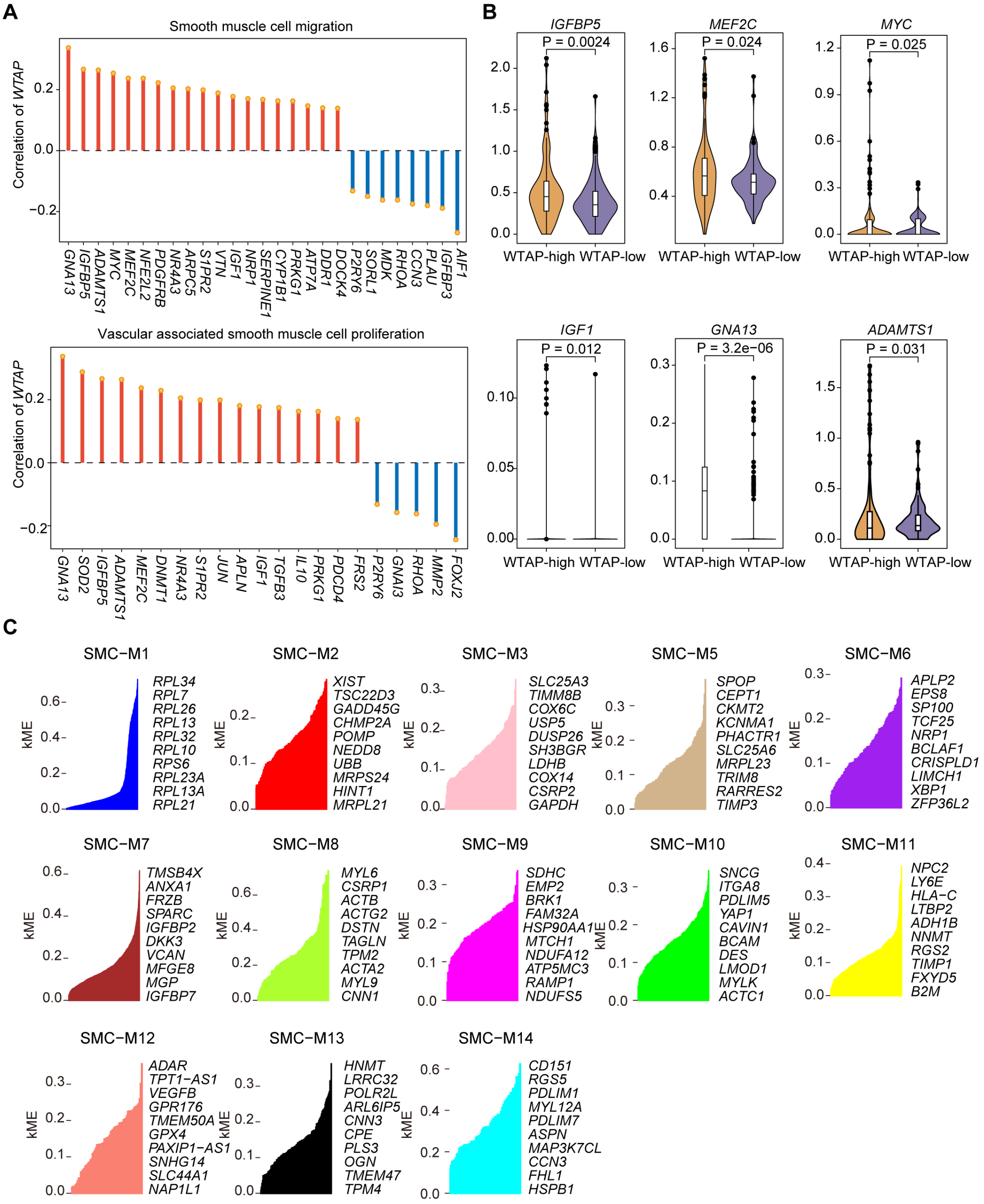

Figure S5. The correlations of *WTAP* and pathway genes, as well as the modules identified in SMCs. (A) The correlations between *WTAP* and genes enriched in SMC migration or proliferation pathways. The red lines represent positive correlation, while the blue lines represent negative correlation. (B) The expression of genes related to SMC migration or proliferation in WTAP-high group and WTAP-low group. (C) Top

10 hub genes ranked by kME in different modules of SMCs.

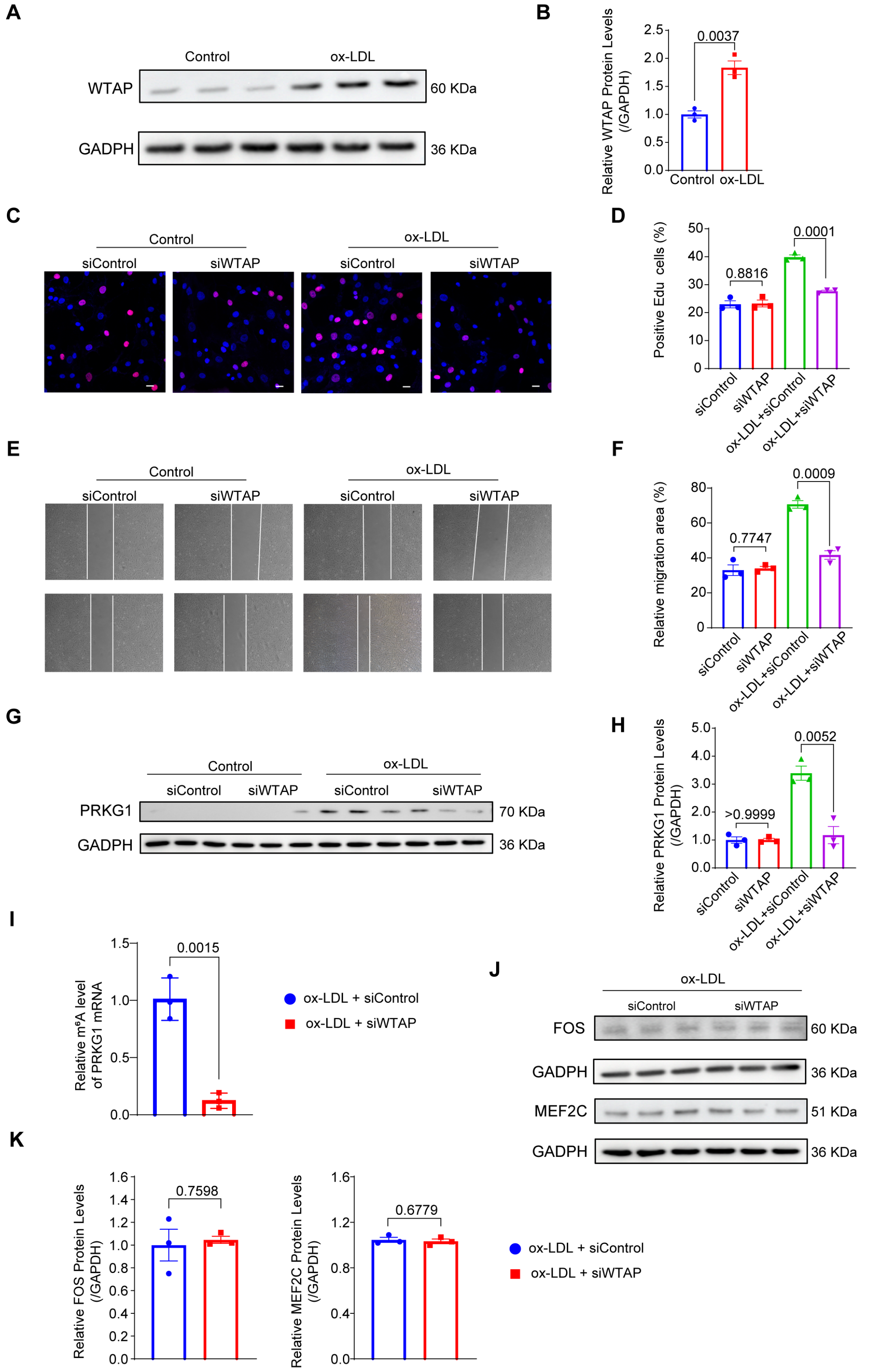

Figure S6. WTAP upregulation in HCASMCs stimulated by ox-LDL is associated

with cell proliferation and migration. (A) Representative western blot images of WTAP levels in control and HCASMCs treated with 100 μg/mL ox-LDL at 48 hours. (B) Quantification of WTAP levels in control and HCASMCs treated with 100 μg/mL ox-LDL at 48 hours (n=3). (C) Cell proliferation was measured by EdU staining. (D) The percentage of EdU-positive HCASMCs (red) was quantified (n=3). Scale bars, 20 μm. (E) HCASMCs migration ability was measured by the Scractch Closure assay. (F) Quantifications of relative migration area of HCASMCs were made (n=3). Scale bars, 500 μm. (G) Representative western blot images of PRKG1 levels in siControl and siWTAP HCASMCs with or without ox-LDL stimulation. (H) Representative quantification of PRKG1 levels in siControl and siWTAP HCASMCs with or without ox-LDL stimulation (n=3). (I) MeRIP-qPCR analysis of PRKG1 m^6A in ox-LDL-induced HCASMCs treated with siControl or siWTAP (n=3). (J) Representative western blot images of FOS and MEF2C levels in HCASMCs transfected with siControl or siWTAP with ox-LDL at 48 hours. (K) Quantification of FOS and MEF2C levels in HCASMCs transfected with siControl or siWTAP with ox-LDL at 48 hours (n=3). Data are presented as mean±SD. T test followed by a Normality test was used.

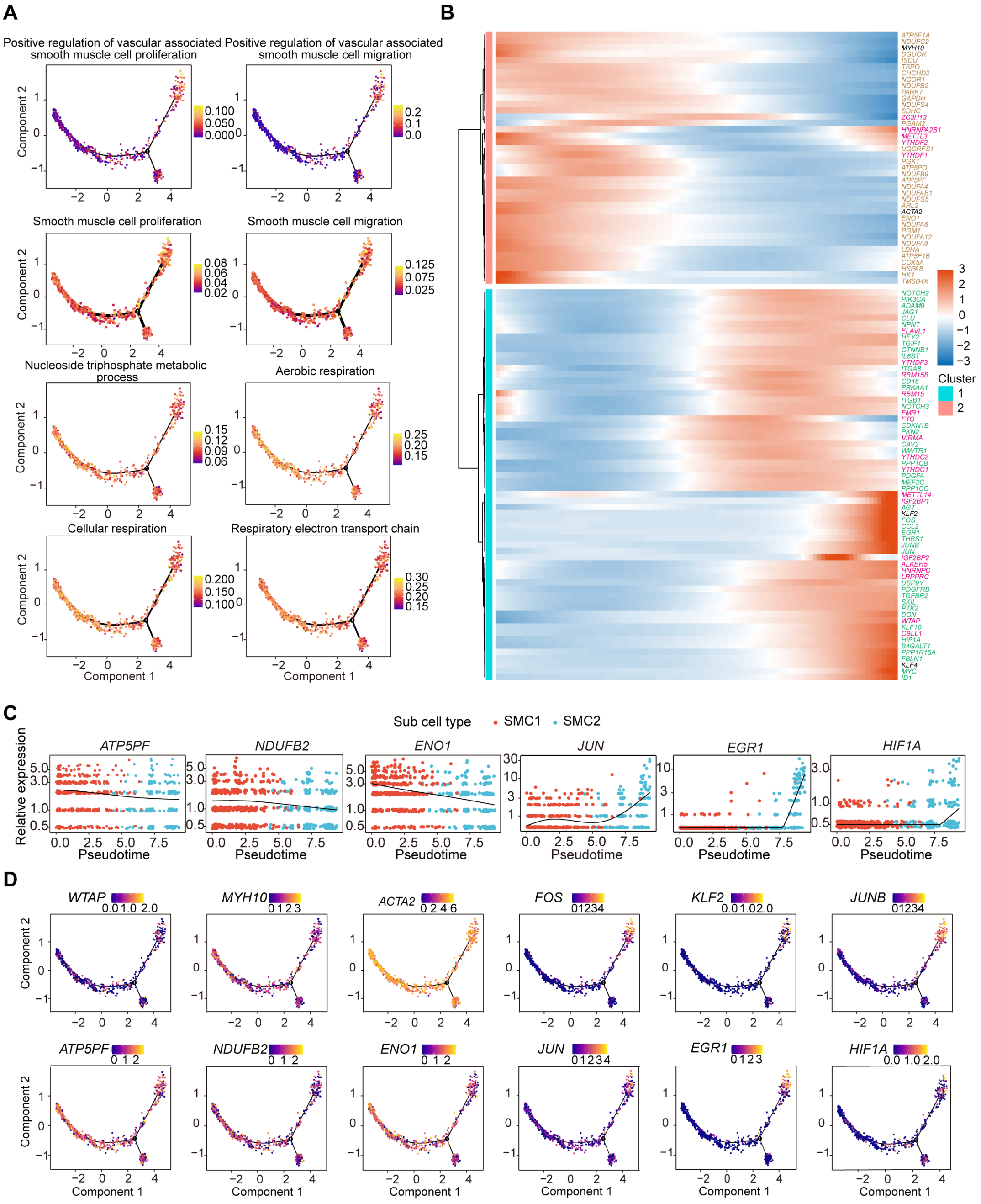

Figure S7. Pseudotime trajectories showing changes of *WTAP* and functional genes during the phenotypic transition of SMCs. (A) Other disease-related pathway AUCell scores enriched along the evolutionary trajectory of SMCs. (B) Heatmap showing changes in gene expressions on pseudotime axis. Genes on the right are colored differently to represent various functional types: brown for energy-related, green for phenotype-related, pink for m^{6}A regulators, and black for marker genes and

reported genes. (C) Dynamic alterations in key functional-related genes during phenotypic transformation. (D) Key functional-related genes expressions enriched along the pseudotime trajectory of SMCs.

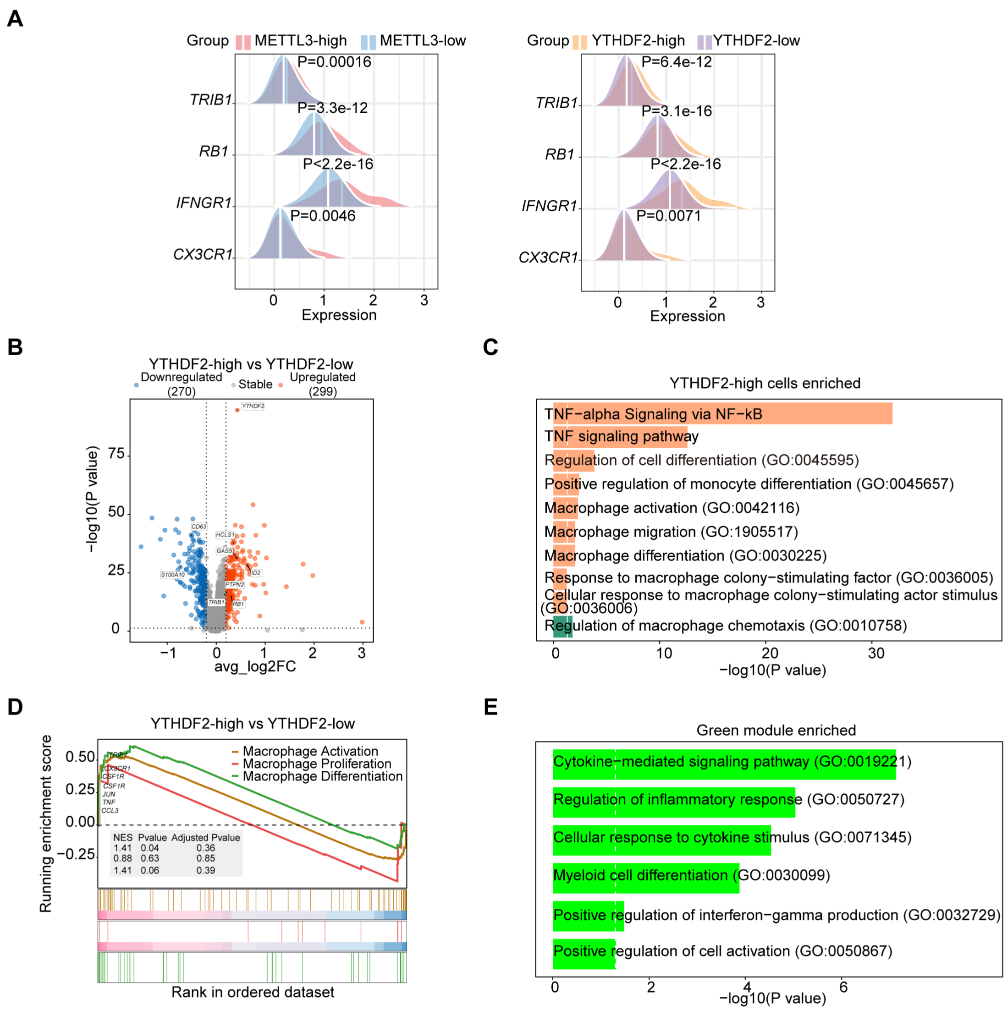

Figure S8. Potential regulation of macrophage genes expressions and function by m⁶A regulators. (A) Comparison of key genes in METTL3-high and METTL3-low groups, as well as in YTHDF2-high and YTHDF2-low groups. The statistical differences between the groups were determined through Wilcoxon rank test. (B) Volcano plot showing DEGs between YTHDF2-high and YTHDF2-low groups. Significant DEGs are shown in orange (upregulation) or blue (downregulation). (C) Disease-related pathways enriched by genes upregulated in YTHDF2-high group. Orange represents upregulation, while green represents downregulation. (D) GSEA showing three macrophage-related pathways. (E) Disease-related pathways enriched by METTL3 co-expressed genes within the green module.

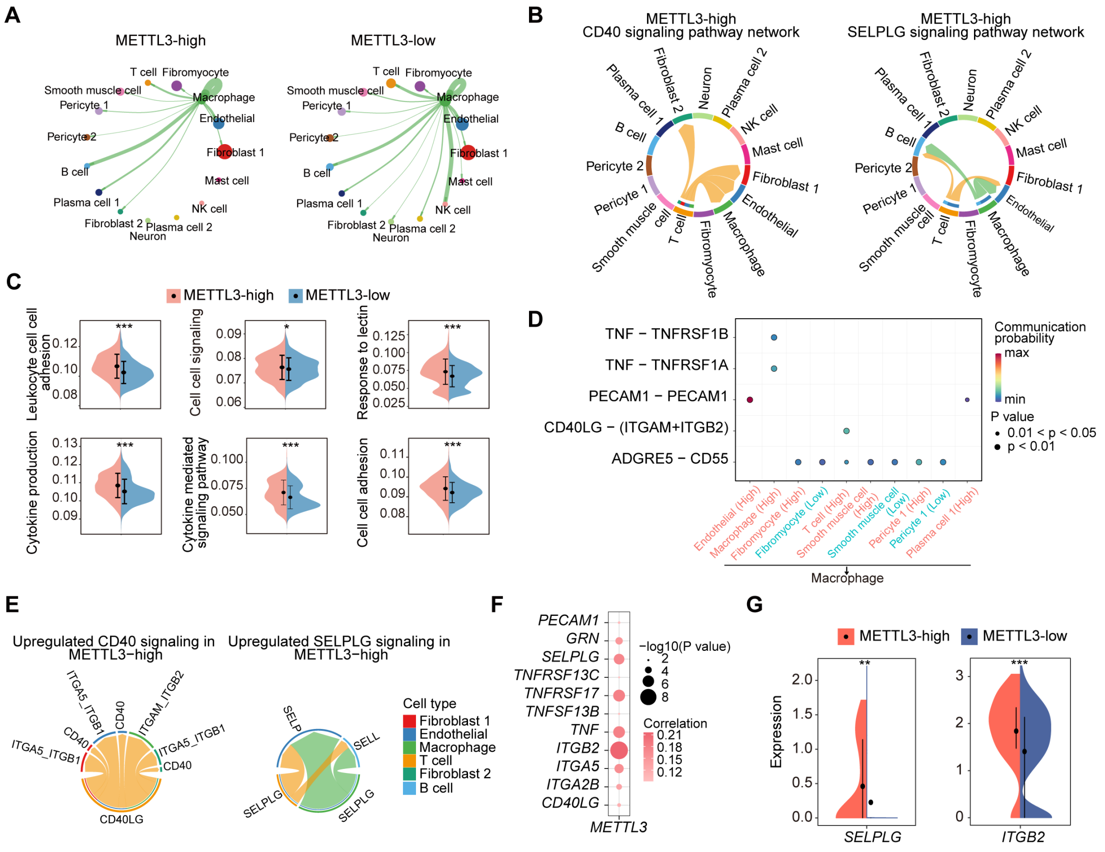

Figure S9. Comparison of macrophages intercellular communication in METTL3-high and METTL3-low groups. (A) Communication strength network of macrophages in METTL3-high and METTL3-low groups. (B) Chord plots showing CD40 and SELPLG signaling pathways of cell-cell communication networks present only in METTL3-high group. (C) The AUCell scores of important signaling pathways in METTL3-high and METTL3-low groups. (D) Comparison of specific ligand-receptor pairs from other cell types to macrophages between METTL3-high group and METTL3-low group. (E) Chord diagram showing the upregulated ligand-receptor pairs of CD40 and SELPLG signaling pathways. (F) Correlations between the expression levels of *METTL3* and ligand-receptor genes. (G) Comparisons of the overall expression of ligand and receptor genes between METTL3-high and METTL3-low groups. The statistical differences between the groups were determined through T test.

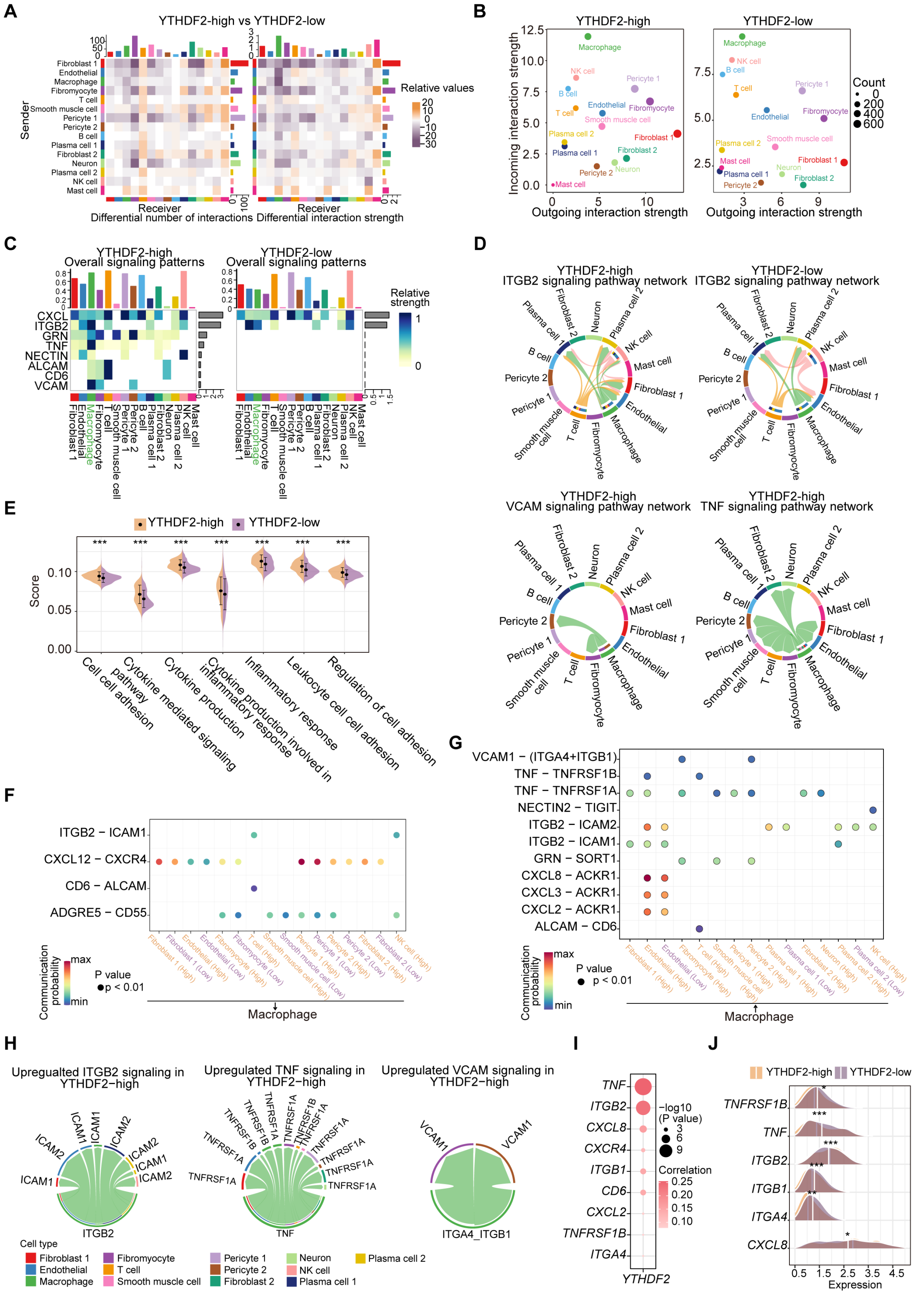

Figure S10. YTHDF2 promotes inflammatory and adhesive functions in macrophages intercellular communication. (A) Heatmaps showing the differences in the number or intensity of interactions between all cell types in YTHDF2-high group

and YTHDF2-low group. Yellow represents an increase in YTHDF2-low group compared to YTHDF2-high group, while purple represents a decrease. (B) Comparison of incoming and outgoing interaction strength in YTHDF2-high group and YTHDF2-low group. (C) Comparison of several signaling pathways in YTHDF2-high and YTHDF2-low groups. (D) Chord plots showing ITGB2 signaling pathway of cell-cell communication networks present in YTHDF2-high and YTHDF2-low groups, along with VCAM and TNF signaling pathways of cell-cell communication networks present only in YTHDF2-high group. (E) The AUCell scores of important signaling pathways in YTHDF2-high and YTHDF2-low groups. The statistical differences between the groups were determined through Wilcoxon rank test. (F) Comparison of specific ligand-receptor pairs from other cell types to macrophages between YTHDF2-high group and YTDHF2-low group. (G) Comparison of specific ligand-receptor interactions from macrophages to other cell types between YTHDF2-high group and YTHDF2-low group. (H) Chord diagram showing the upregulated ligand-receptor pairs of ITGB2, TNF and VCAM signaling pathways. (I) Correlations between the expression levels of *YTHDF2* and ligand-receptor genes. (J) Ridge plot showing the ligand-receptor pair genes that are significantly expressed in YTHDF2-high group. The statistical differences between the groups were determined through T test.

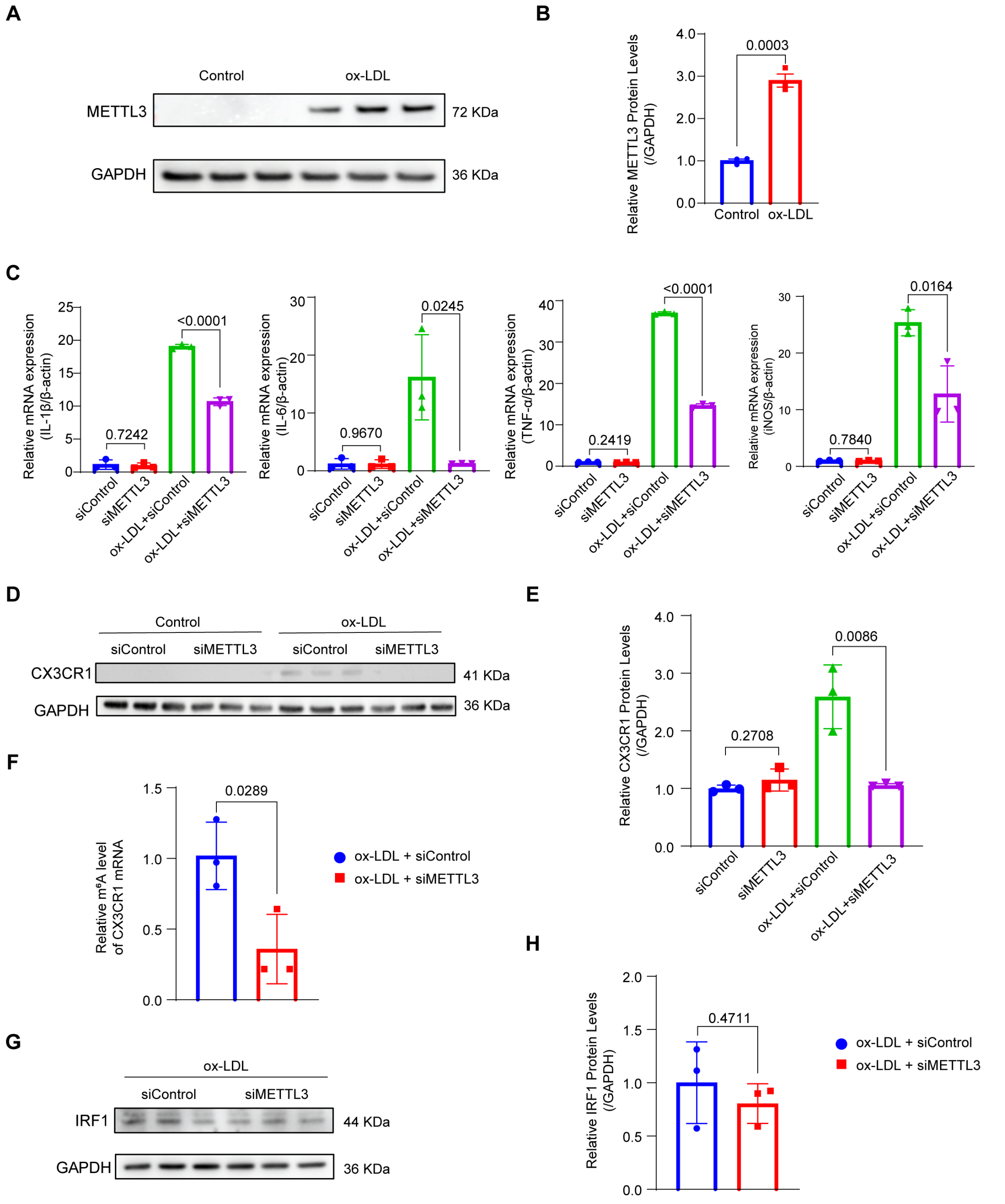

Figure S11. METTL3 aggravates macrophages-mediated inflammatory response.

(A) Representative western blot images of METTL3 levels in control and THP-1 treated with 150 μg/mL ox-LDL at 12 hours. (B) Quantification of METTL3 levels in control and THP-1 treated with 150 μg/mL ox-LDL at 12 hours (n=3). (C) RT-qPCR quantification of IL-1β, IL-6, TNF-α and iNOS levels in control and THP-1 treated with 150 μg/mL ox-LDL at 12 hours (n=3). (D) Representative western blot images of

CX3CR1 levels in siControl and siMETTL3 THP-1 with or without ox-LDL stimulation (n=3). (E) Representative quantification of CX3CR1 levels in siControl and siMETTL3 macrophages with or without ox-LDL stimulation (n=3). (F) MeRIP-qPCR analysis of CX3CR1 m^6A in ox-LDL-induced macrophages treated with siControl or siMETTL3 (n=3). (G) Representative western blot images of IRF1 levels in siControl and siMETTL3 THP-1 with ox-LDL stimulation (n=3). (H) Quantification of IRF1 levels in macrophages transfected with siControl or siMETTL3 with ox-LDL (n=3). Data are presented as mean±SD. T test followed by a Normality test was used.
